# Avian species richness and tropical urbanization gradients: Effects of woodland retention and human disturbance

**DOI:** 10.1002/eap.2586

**Published:** 2022-06-19

**Authors:** Phakhawat Thaweepworadej, Karl L. Evans

**Affiliations:** ^1^ School of Biosciences, The University of Sheffield Sheffield UK

**Keywords:** avian assemblage, cities, exotic species, habitat creation, habitat fragmentation, habitat restoration, spatial configuration, species turnover

## Abstract

Urbanization is a major driver of tropical biodiversity loss. In temperate regions avian species richness–urbanization intensity relationships typically exhibit unimodal patterns, with peak richness at intermediate urbanization levels. In tropical regions, the form of such relationships and the extent to which they are moderated by patches of seminatural habitat are unclear. We address these questions in Bangkok, Thailand (one of the largest and most rapidly expanding tropical mega‐cities) and generate conservation recommendations for tropical biodiversity in urban locations. We use repeated point count surveys at a random location, and the largest available woodland patch, in 150 1 km × 1 km grid cells selected along the urbanization gradient. Woodland patches support higher species richness compared with randomized locations (except for non‐natives), and avian species richness declines linearly with increasing urbanization. The contrast with unimodal patterns in temperate regions is probably driven by divergent patterns of habitat heterogeneity along tropical and temperate urbanization gradients. Moreover, we provide novel evidence that retaining patches of urban woodland moderates adverse impacts of urbanization on avian species richness. For most species groups, the benefits of woodland increase as urbanization intensifies, despite such woodland patches being very small (mean of 0.38 ha). Avian species richness in woodland patches is maximized, and community composition less similar to that in randomized locations, when woodland patches are larger and visited by fewer people. Assemblages of forest‐dependent species (which provide additional ecological functions) have higher richness, and are less similar to those in randomized locations, in patches of woodland with higher tree species richness and biomass. Finally, species richness in randomized sites is greatest when they are closer to woodland patches, and such assemblages more closely resemble those of woodland sites. Our work highlights four strategies for tropical urban bird conservation: (1) conserving woodland patches across the urbanization gradient regardless of patch size, (2) improving the quality of existing woodland by increasing tree biomass and diversity, (3) creating additional woodland that is well distributed throughout the urban area to minimize effects of habitat isolation and (4) reducing human disturbance, especially in areas of the highest habitat quality, while ensuring that the benefits of connecting people to nature are realized in other locations.

## INTRODUCTION

Urbanization generates marked landscape alteration, and is a key driver of environmental changes (d'Amour et al., 2017; Deng et al., 2015; Wei & Ye, 2014) and biodiversity loss (Aronson et al., 2014; Rebelo et al., 2011; Sol et al., 2014). Species vary in their ability to cope with landscape alteration induced by urbanization and accompanying selection pressures, which increase in magnitude along the urbanization gradient (Grimm et al., 2008; Isaksson, 2018; Johnson et al., 2015). Specialist species are therefore most likely to be absent from urban areas and typically exhibit the greatest declines in population densities (Callaghan et al., 2019; Evans et al., 2011). Consequently, intensively urbanized locations support a limited set of native species compared with less urbanized locations, contributing to biotic homogenization (Colléony & Shwartz, 2020; McKinney & Lockwood, 1999; Zeeman et al., 2017).

The precise pattern in which species richness changes along urbanization gradients is, however, rather variable across taxonomic groups and geographical locations (McKinney, 2008). Among birds, for example, in temperate regions species richness tends to exhibit a unimodal/hump‐shaped pattern along urbanization gradients with maximum species richness in suburban areas with intermediate levels of urbanization intensity (Blair, 2004; Crooks et al., 2004; Tratalos et al., 2007; Luck & Smallbone, 2010; McKinney, 2002; Smith & Wachob, 2006; Vignoli et al., 2013). Such patterns are usually attributed to greater habitat diversity in suburban locations that promote local species richness, even though some specialists are excluded from such locations (Blair & Johnson, 2008; McKinney, 2002; Tratalos et al., 2007). In contrast, in tropical regions avian species richness may decline in a linear manner along urbanization gradients (Bhatt & Joshi, 2011; Leveau et al., 2017; Reis et al., 2012), but hump‐shaped patterns have also been reported (Leveau, 2019) and more studies from tropical regions are required (Marzluff, 2001, 2017). The mechanisms generating linear declines in species richness in tropical regions (Chamberlain et al., 2017; Leveau et al., 2017; Reis et al., 2012), rather than hump‐shaped curves, are unclear but may be due to differences in urban form and landscape characteristics with less urbanized locations in tropical regions containing more seminatural habitat and less intensively managed agricultural land compared with those in temperate regions.

Regardless of the precise pattern of declines in species richness, it is clear that intensely urbanized locations have lower biodiversity compared with locations that are less urbanized (Sol et al., 2014). These lower levels of biodiversity in urbanized locations have some important consequences for conservation including the direct loss of biodiversity (McDonald et al., 2008, 2013; Sodhi et al., 2010), and reduced opportunities for city dwellers to engage with nature that can deliver well‐being benefits (Coldwell & Evans, 2018; Schebella et al., 2019) and increase their appreciation of the natural world and support for conservation (Clergeau et al., 2001; Coldwell & Evans, 2017; Lo & Jim, 2010). Urban areas with higher levels of biodiversity may also generate more ecosystem services, for example, food provision (Orsini et al., 2014; Speak et al., 2015), pollination (Baldock et al., 2019), carbon sequestration by urban trees (Agbelade & Onyekwelu, 2020), etc. There is therefore considerable interest in how to increase biodiversity in urban environments.

Urban bird diversity is positively associated with the size of urban green areas (Kaushik et al., 2021; Sorte et al., 2020). Options for enhancing urban biodiversity by increasing the amount of urban green space in currently urbanized locations are typically limited and expensive, although there is some potential for retrofitting green walls and roofs (Belcher et al., 2019; Collins et al., 2017; Orsini et al., 2014; Wang et al., 2017) or directly converting impervious surfaces to green space (Qian et al., 2015). Cost‐effective opportunities to enhance urban biodiversity are more likely to arise through improving the quality of existing green space by changing management practices, or replacing types of green space that support limited amounts of biodiversity with habitats that support a wider range of species (Aronson et al., 2017; Threlfall et al., 2017). Such habitat replacement schemes often focus on enhancing the environmental quality of urban grasslands through converting intensely mown grassland to systems that resemble species rich meadows (Norton et al., 2019). Woodland areas also play a major role in retaining biodiversity in urban areas, especially for avian biodiversity (Melles et al., 2003; Pellissier et al., 2012; Plummer et al., 2020). Observed positive correlations between woodland cover and biodiversity may arise primarily because woodland increases at the expense of the amount of urban land, that is, higher biodiversity in areas with more woodland may be simply due to lower levels of urbanization in such locations (Filloy et al., 2019). Alternatively, increasing the amount of woodland in highly urbanized areas could mitigate some of the adverse impacts of urbanization on biodiversity, in which case the form of the relationships between biodiversity and urbanization intensity would be modified by the amount of woodland cover. This will, however, be context dependent and vary depending on the proportion of forest‐dependent species in a landscape's avifauna, and the sensitivity of those species to urban stressors. We are not aware of any tests of such moderating impacts of urban woodland, but their occurrence would point to the potential effectiveness of increasing woodland cover in urban areas through habitat restoration/creation schemes that could promote higher levels of avian biodiversity within towns and cities.

The quality of urban woodlands is likely to play a role in their impact on urban bird assemblages. Woodlands that contain larger trees are likely to provide more resources, such as fruit or phytophagous insects, due to allometric relationships and other resources, for example cavities that are required as nest sites, may occur exclusively in larger older trees (Harper et al., 2005). Large trees are therefore keystone structures for urban biodiversity (Stagoll et al., 2012). A greater diversity of resources may also be provided by woodlands with a greater mix of tree species, and due to interspecific variation in flowering and fruiting times, may provide greater stability of resources. Although more studies are required it is notable that urban avian species richness responds positively to the density (Barth et al., 2015; Fontana et al., 2011), richness (Ferenc et al., 2014; Paker et al., 2014), and size of trees (MacGregor‐Fors, 2008; Stagoll et al., 2012) in urban woodlands. Landscape factors are also likely to play a role in determining the composition of avian assemblages in urban woodlands as woodland specialists may be more reluctant to travel through the urban matrix to cross gaps between woodland patches (Watson et al., 2005). Finally, evidence is accumulating that human disturbance can adversely impact avian territory establishment and species richness in woodlands (Bötsch et al., 2017), and some studies also report such effects in urban locations (e.g., MacGregor‐Fors & Schondube, 2011).

Our study has four core objectives. We first test the hypothesis that in tropical regions avian biodiversity declines linearly with urbanization intensity, rather than exhibiting the unimodal pattern typically exhibited in temperate regions. Second, we test if woodland cover along the urbanization gradient can modify the form of these relationships; we do so by comparing how species richness along the urbanization gradient changes when sampling locations with typical conditions for a given urbanization intensity (randomly selected locations) and when sampling nearby woodland patches embedded within the same urban matrix. Third, we test how environmental characteristics of the sampling locations (relating to human disturbance, woodland quality and habitat fragmentation and isolation) influence avian species richness, and fourth test how turnover in species composition between randomly selected and wooded locations varies with these environmental characteristics. Our results inform understanding of how urbanization in tropical regions impacts biodiversity, and the potential of urban woodland to minimize adverse impacts, and how to design such woodlands to maximize biodiversity benefits.

## METHODS

We use a southeast Asian case study (Bangkok, Thailand) as much of the urban development in this region has occurred in its biodiversity hotspots (as defined by Myers et al., 2000), driving considerable biodiversity loss (Hughes, 2017; Sodhi et al., 2004), and future urbanization is predicted to follow a similar pattern (Seto et al., 2012). Bangkok provides a particularly suitable case study as it is one of the most rapidly urbanizing mega‐cities in this region, and globally (Estoque & Murayama, 2015; Hokao et al., 2012; Song et al., 2021).

### Study area

**FIGURE 1:**
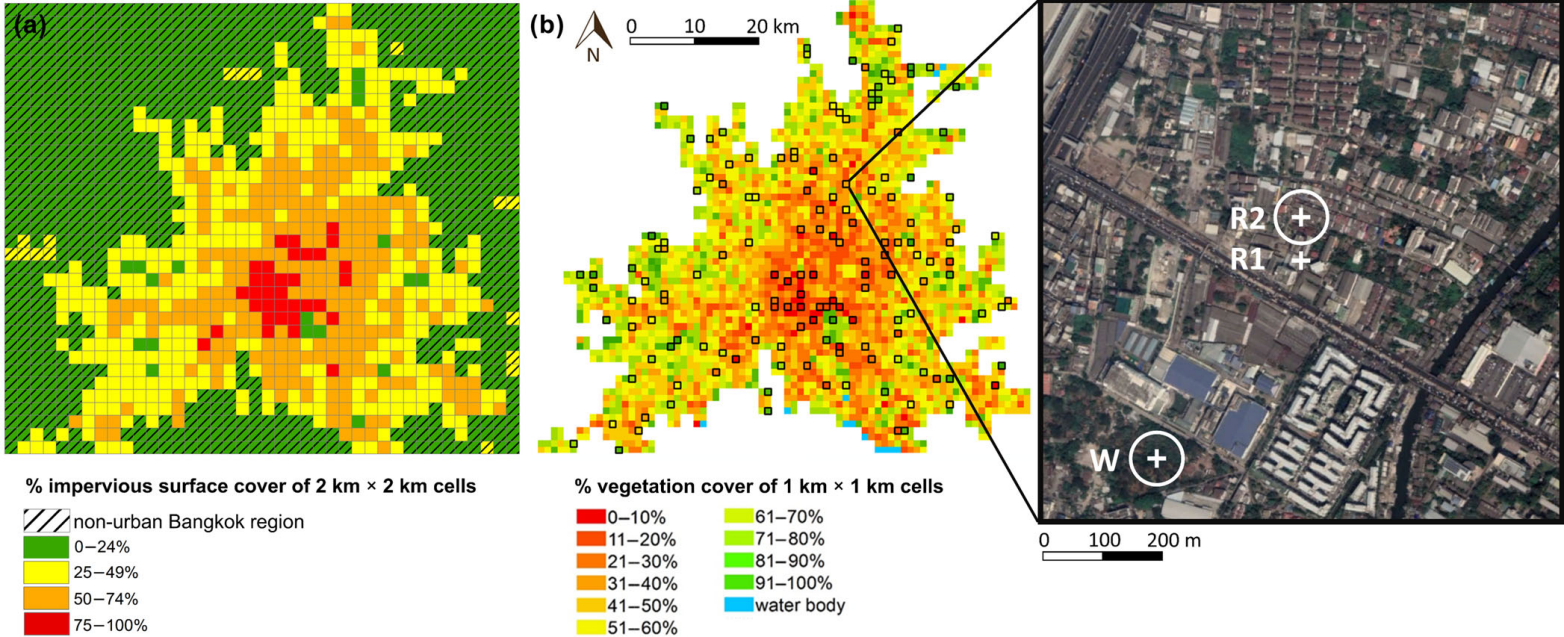
(a) Landcover map of the Bangkok study region showing percentage impervious surface cover of 2 km × 2 km grid cells used to define the urban study region. Grid cells with ≥25% impervious surface cover that were isolated from the main urban region or had <25% impervious surface cover were not classified as part of urban Bangkok. (b) The percentage vegetation cover of 1 km × 1 km grid cells across the urban study region. Grid cells outlined in black represent the 150 cells selected for sampling using random stratification across each category of percentage vegetation cover (15 sampling grid cells each). The inset map shows an example 1 km × 1 km grid cell with the location of the randomized point (R2), which is located at the nearest accessible point to the north of the grid cell's center (R1) as this is inaccessible to the observer. The woodland sampling plot (W) is located in the largest area of woodland within the focal grid cell. White circles show point counts' 50 m sampling radii

We defined the urban area of Bangkok as the 2 km × 2 km cells with at least 25% impervious surface cover (following Bonnington et al., 2014). To determine the boundaries of this region we first delimited a 5600 km^2^ area (1400 2 km × 2 km cells) that was centered on Metropolitan Bangkok and the surrounding provinces (Appendix S1: Figure S1). We used high resolution imagery from Google Earth that was captured in either 2017 or 2018 and following Evans et al. (2009), we estimated the percentage of each 2 km × 2 km grid cell that comprised impervious surfaces using 100 uniformly distributed sampling points within each grid cell. This delimited a study region of 2628 km^2^ (Figure 1a). Google Earth uses a mixture of aerial imagery from a variety of satellites and planes, and is increasingly used as a source of landcover data in ecological and remote sensing studies, especially when alternative data layers are unavailable (Liang et al., 2018).

### Selecting survey sampling points

For each 1 km × 1 km grid cell within our sampling region we calculated the percentage land cover for each of nine categories (impervious surface cover, trees, grassland, rice fields, salt pans, green roofs, bare ground, construction site and waterbody) using our sampling grid (please refer to section “*Study area*”). We then classified each 1 km × 1 km grid cell within the study region into one of 10 categories of urbanization intensity based on their percentage vegetation cover (i.e., combining trees, grasslands, and rice fields; 0%–10%, 11%–20%, …, 91%–100%) with more urbanized locations having less vegetation cover. We use a smaller 1 km × 1 km grid cell to define our focal urbanization gradient than the spatial grain used to delimit the urban study area due to the very large spatial extent of the Bangkok urban area, and the effects of spatial scale on delimiting urban and non‐urban areas (please refer to e.g., Moll et al., 2019). Our use of a 1 km spatial grain for defining the urbanization gradient matches that of many other urban ecology studies assessing biodiversity responses to urbanization, especially in temperate regions (e.g., Bonnington et al., 2014), which helps compare our tropical case study to previous research.

We used random stratification to select 15 1 km × 1 km grid cells within each percentage vegetation cover category, resulting in a total of 150 sampling cells spread along a gradient of urbanization intensity. Two sampling points were located within each grid cell. The first sampling point (referred to as the randomized point) was located at the center of each randomly selected grid cell. It can therefore fall in any landcover type and represents typical conditions of grid cells at similar levels of urbanization intensity. When the center of a grid cell was inaccessible to the observer, we used the nearest accessible location (Figure 1b). The second sampling point was located at the center of the largest accessible patch of trees/woodland (referred to as the woodland point). Comparing the avian assemblages across these two types of sampling locations enables us to assess if woodland cover modifies the effects of urbanization intensity on tropical urban avian biodiversity.

### Field surveys

We conducted bird surveys using point counts with a 50 m survey radius and 15 min duration. This is at the upper end of the range of durations typically used for avian point counts (Bibby et al., 2000) because longer durations are preferable when (1) surveying more diverse communities (Turner, 1996), (2) travel time between survey locations is long (Vergara et al., 2010), typically 30 min between survey grid cells in our study, and (3) study objectives require greater focus on reducing false absences than reducing double counting of individuals. This is the case in our study as our focus is on habitat influences on species richness and composition data, and false absences distort models of such habitat associations (Gu & Swihart, 2004). We recorded all birds heard or seen during the point count. Surveys took place from 6:30 AM until 12:00 PM (noon). We used a rangefinder (Viking Compact Laser Rangefinder) to ensure that detected birds were within the 50 m radius of the survey point. To capture seasonal variation, we visited each sampling point three times during March to July 2018 (first visit 12 March to 28 April; second visit 2 May to 11 June; third visit 12 June to 25 July). The first visit captures the period when wintering and passage migrants are present in the Bangkok region, while the second and third visits overlap with the breeding season of most species in the region. Within each sampling period we visited survey locations in an order that was unbiased with regard to their urbanization intensity. This was achieved by visiting approximately four sampling cells each day that were within the same part of the city (to reduce travel time), but represented different urbanization categories, and that were also in different urbanization categories to those visited the following day, and so on. We visited the survey cells that were surveyed on the same day in a different order across the three survey rounds.

We recorded the number of people detected in the sampling area during each point count to generate an index of human disturbance (calculated as mean number of people across three visits). The abundance of most resources provided by trees, such as fruits, flowers, or phytophagous insects, is mainly determined by tree biomass and therefore larger trees contribute disproportionately. We identified all trees within the radius of each point count with at least 25 cm diameter at breast height (dbh) to species, and recorded their dbh and height. We defined large trees as those with ≥70 cm dbh (following Slik et al., 2013). We then calculated the aboveground biomass of surveyed trees using allometric equations from Chave et al. (2005), with wood density data obtained from Global Wood Density Database (Zanne et al., 2009), and scaled to tonnes/ha to provide an index of the density of tree biomass within each point count area.

### Species groups

We split the total avian assemblage into the following subgroups: native and non‐native to the Bangkok area (because assessing if urbanization promotes non‐native species is of considerable interest in the urban ecology literature; Marzluff, 2001; Tomasevic & Marzluff, 2017), residents and migrant non‐breeders (because urbanization has been hypothesized to adversely affect migrants relative to residents; Biamonte et al., 2011), and forest and non‐forest‐dependent species (because these are expected to respond differently to woodland). We defined native and seasonal status using classifications from Round and Gardner (2008) and forest dependency status based on BirdLife International (2019). Species with “high forest dependency” are forest specialists that mainly occur in undisturbed forests and are rarely found in degraded forest habitats, “medium forest‐dependent” species are mainly found in undisturbed forest but also regularly occur in degraded forest habitat such as forest edge and secondary forest, and “low forest‐dependent” species are mostly generalists and well adapt to live and breed in disturbed habitats (Buchanan et al., 2008, 2011). We classified species defined by BirdLife as “does not normally occur in forest” and “low forest‐dependent species” as non‐forest‐dependent species, and included species defined by BirdLife as “medium forest‐dependent species” as forest‐dependent species (no species with a high forest dependency were detected in our surveys; please refer to [https://doi.org/10.6084/m9.figshare.16557021]).

### Landscape scale data

We obtained percentage impervious surface cover and tree cover in each 1 km × 1 km grid cell from our landcover classifications (described above). We defined urban woodland patches as areas with contiguous tree canopy cover that were at least 0.02 ha. We measured woodland size by delimiting the edge of continuous tree canopy cover and measuring the resultant area using Google Earth (Google Earth Pro v7.3.2). We measured the straight‐line distance between the randomized and woodland survey points, and the distance between the randomized point and the edge of the nearest woodland patch.

### Data analyses

We conducted all analyses using R version 3.4.2 (R Core Team, 2017), with the name of the R package used for each analysis provided below in italics, and checked diagnostic plots to confirm model assumptions were met.

To meet our first objective of testing if species richness declines linearly with increasing urbanization intensity or peaks at intermediate levels of urbanization intensity, we model species richness as a function of impervious surface cover using linear and quadratic terms. We present models separately for randomized and woodland points. We do so as the alternative for constructing models that pool data across both types requires constructing models with multiple interaction terms (due to use of linear and quadratic terms) that complicates interpretation of model outputs. We construct these models for total species richness, native species richness, non‐native species richness, resident species richness, migrant non‐breeder species richness, non‐forest species, and forest‐dependent species. All models were fitted with Gaussian error structure (identity link), except migrant non‐breeder species richness (both location types) and non‐forest species richness at the woodland points that were fitted with Poisson error structure (log link) in the *stats* package, and non‐forest species richness at the randomized points that was fitted using a negative binomial model in the *MASS* package. We determined error structure based on the models' AIC_c_ values (Appendix S2: Table s1), and checked for overdispersion in Poisson models using the *DHARMa* package (Appendix S2: Table S2). We determined *a priori* that evidence for a quadratic relationship would be provided if the quadratic model's AIC_c_ value was ≥2 points lower than the linear model, and the *p*‐value of the quadratic term was statistically significant. In all models, we calculated the model (or partial) *R* ^2^ values using the *rsq* package that employs the methodology described by Zhang (2017).

These models confirmed that all changes in species richness along the urbanization gradient followed a linear pattern (please refer to section “*Results*”). This enabled us to test if woodland cover modified the slope of the relationship between species richness and urbanization intensity using a unified model that pooled data from woodland and randomized locations. We therefore modeled species richness as a function of impervious surface percentage, location type (i.e., randomized or woodland point), and grid cell ID (random effect) and the interaction term between impervious surface percentage and location type (i.e., randomized or woodland point). We excluded three grid cells in which the randomized points were located within the grid cell's largest woodland from this analysis. Again, we fitted our models with Gaussian error structure (identity link), except migrant non‐breeder species richness (both location types) and non‐forest species richness at the woodland points that were fitted with Poisson error structure (log link), and non‐forest species richness at the randomized points that was fitted using a negative binomial model. We conducted Moran's I tests on the residuals from all our models using the *ape* package. This uses an inverse distance matrix approach rather than a nearest neighbor approach, which uses correlograms to assess the optimum lag distance at which to define the nearest neighbor; implemented using *letsR* package. The inverse distance matrix approach is frequently deployed in urban ecology studies (Cubino et al., 2019; Kurylo et al., 2020) and is advantageous in our study system as correlograms indicated no clear lag distance at which spatial autocorrelation peaks (Appendix S2: Figures S1–S5). Our checks did not detect any significant residual spatial autocorrelation in species richness models at the randomized points, but did so for some species richness metrics at the woodland points, but Moran's I values were consistently low (≤0.022; Appendix S2: Table S3). Moreover, parameter estimates in models that did and did not take spatial correlation structure into account were extremely similar (Appendix S2: Table S4). Therefore, in the main manuscript, we only report results from non‐spatial models.

We assessed factors influencing bird species richness by performing multiple regression analyses for bird species richness with landscape characteristics and ecological attributes of the wooded habitat, separately for randomized and woodland points. We did so using a full model approach following the advocacy of Whittingham et al. (2006). Bird species richness at the randomized points was modeled with percentage impervious surface cover of the grid cell, distance to the nearest woodland, mean number of humans, tree species richness, total aboveground tree biomass, and aboveground tree biomass of large trees (Table 1). We used a near identical set of explanatory variables to predict bird species richness at the woodland points, with the exception being using size of the sampled woodland instead of distance to the nearest woodland. We fitted models with Gaussian error structure and identity link for total species richness, native species richness, non‐native species richness, resident species richness, and forest‐dependent species richness. We fitted models with Poisson error structure and log link for migrant non‐breeder species richness, and non‐forest species richness; we selected error structures that generated the best fit to the data based on AIC_c_ values (Appendix S2: Table S1). Our models were not unduly influenced by overdispersion (assessed using *DHARMa* package; Appendix S2: Table S2).

**TABLE 1:** Description of predictor variables used in multiple regression models of bird species richness and species turnover (Jaccard's dissimilarity index) in bird communities in woodland and randomized sampling points. Central values are means for impervious surface percentage and distance from randomized plot to the sampled woodland, and the median for predictors with natural log transformation (used to reduce the skew in predictor distributions)

Predictor variables	Units	Central value	Range	Transformation
Landscape scale				
% impervious surface cover of grid cell	%	46.86	0–96.00	–
Distance from random plot to the nearest woodland	m	15.00	0–445.00	ln (x + 1)
Distance from random plot to the sampled woodland	m	245.00	5.00–540.00	–
Size of the sampled woodland	ha	1.14	0.11–87.71	ln (x)
Point count scale (randomized point)				
Mean no. humans	people	11.40	0–121.67	ln (x + 1)
Tree species richness	species	6.00	0–22.00	ln (x + 1)
Total aboveground tree biomass	t/ha	7.99	0–89.89	ln (x + 1)
Aboveground tree biomass of large trees	t/ha	0	0–82.41	ln (x + 1)
Point count scale (woodland point)				
Mean no. humans	people	0.67	0–89.67	ln (x + 1)
Tree species richness	species	9.00	1.00–30.00	ln (x)
Total aboveground tree biomass	t/ha	29.83	2.86–144.92	ln (x)
Aboveground tree biomass of large trees	t/ha	0	0–103.98	ln (x + 1)
Point count scale (absolute difference between locations)				
Mean no. humans	people	4.33	0–118.67	ln (x + 1)
Tree species richness	species	5.00	0–24.00	ln (x + 1)
Total aboveground tree biomass	t/ha	15.19	0.36–136.36	ln (x)
Aboveground tree biomass of large trees	t/ha	2.34	0–103.98	ln (x + 1)

Use of the “vif” function in the *car* package (Fox & Weisberg, 2019) revealed that none of our species richness models was unduly influenced by collinearity (Variance Inflation Factors [VIFs] were consistently below the threshold above which collinearity becomes a concern; VIF > 10; Dormann et al., 2013) (Appendix S2: Table S5). Moran's I tests detected significant residual spatial autocorrelation in some models of species richness metrics as a function of habitat features, but the Moran's I values were again consistently low (≤0.033; Appendix S2: Table S6). Comparison between models with and without taking spatial correlation structure into account revealed very limited differences in coefficient estimates and standard errors and we therefore only report results from non‐spatial models in the main manuscript (Appendix S2: Table S7).

To further understand how patches of woodland influence species composition of urban bird communities along the urbanization gradient, we quantified differences in community composition between the randomized and woodland survey points using Jaccard's dissimilarity index (1 − Jaccard's similarity index; Chase & Leibold, 2002). We calculated Jaccard's dissimilarity index for total species richness, native species richness, resident species richness, and forest dependency categories, but not non‐native species and migrant non‐breeders as few species within each of these groups were recorded in total (non‐native species: 5 species; migrant non‐breeders: 36 species) and at individual point count locations (non‐native species: randomized point median = 2 (range 0–3); woodland point median = 2 (range 0–3); migrant non‐breeders: randomized point median = 0 (range 0–4); woodland point median = 1 (range 0–6)). Dissimilarity indices were then modeled again using a full model approach, as a function of landscape scale variables and measures of “environmental roughness” (*sensu* Gaston et al., 2007) that is, the difference in environmental conditions between the two sampling locations. The complete list of predictor variables is percentage impervious surface cover (within the 1 km grid cell in which the pair of points is located), distance from randomized point to the nearest woodland, distance from randomized point to the sampled woodland, size of the sampled woodland, absolute difference in mean number of humans, absolute difference in tree species richness, and absolute difference in total aboveground tree biomass (Table 1). Variance Inflation Factors for species turnover models were again consistently lower than the threshold, indicating no influence of multicollinearity among our predictors in species turnover models (Appendix S2: Table S5). Moran's I tests did not detect significant autocorrelation in the residuals of our models of Jaccard's dissimilarity (Appendix S2: Table S6).

## RESULTS

### General description of the avifauna

We detected a total of 142 bird species across the 300 point count locations during three visits between March and July 2018. These comprised 99 residents, 36 migrant non‐breeders, five passage migrants, and two migrant breeders (please refer to [https://doi.org/10.6084/m9.figshare.16557021]). The vast majority of species are native to the Bangkok area (137 species; 96.5%) with only five species (3.5%) being locally non‐native (Zebra Dove *Geopelia striata*; native to southern Thailand, Alexandrine Parakeet *Palaeornis eupatria*; native to northwestern and western Thailand) or nationally non‐native (Rock Pigeon *Columbia livia*, Java Sparrow *Lonchura oryzivora*, Rose‐ringed Parakeet *Psittacula krameri*). While two of these species are of global conservation concern, they had very low rates of occurrence (Alexandrine Parakeet *Palaeornis eupatria*, near‐threatened, 1.3% of grid cells; Java Sparrow, endangered, 1.3% of grid cells; BirdLife International, 2019), as did Rose‐ringed Parakeet *Psittacula krameri* (2% of grid cells), contrasting with the much more widespread Rock Pigeon *Columbia livia* (91.3% of grid cells) and Zebra Dove *Geopelia striata* (100% of grid cells). Non‐forest‐dependent species comprised 71.1% (101 species) of the detected species, with forest‐dependent species comprising the remaining 28.9% (41 species).

### Objective 1: Shape of species richness–urbanization intensity relationships

**FIGURE 2:**
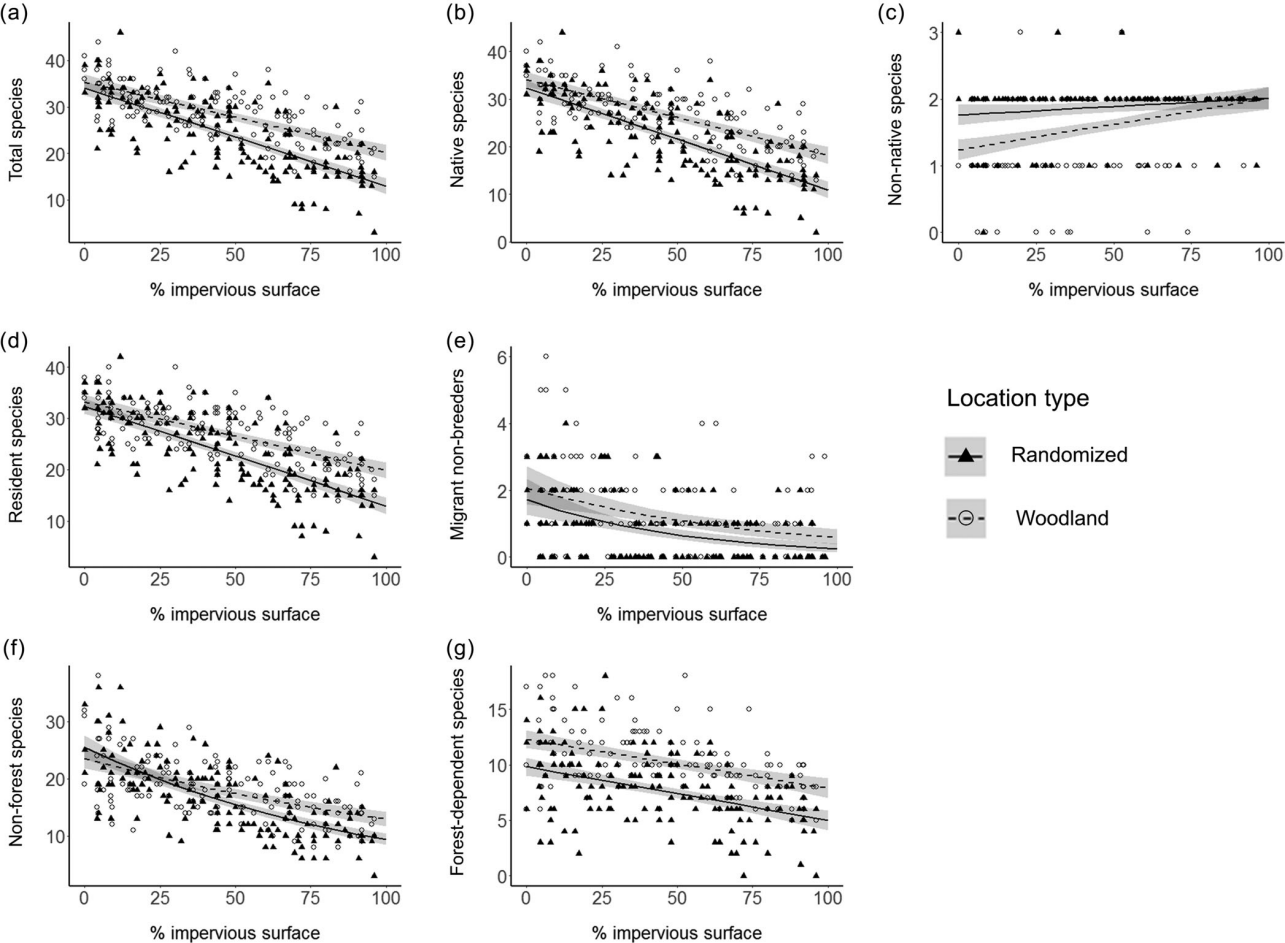
Bird species richness; (a) total species richness, (b) native species richness, (c) non‐native species richness, (d) resident species richness, (e) migrant non‐breeder species richness, (f) non‐forest species richness, and (g) forest‐dependent species richness as function of impervious surface percentage comparing between randomized (filled triangle and solid line) and woodland point (open circle with dashed line). Fitted lines indicate predicted values from linear mixed model with Gaussian error structure (a–d, g) and generalized linear mixed model with Poisson error structure (e, f) with shades representing confidence intervals (please refer to Table 3)

For all our species richness metrics the quadratic models never met our criteria of having a delta AIC_c_ value ≤2 relative to the linear model, and a statistically significant quadratic term (Table 2). These criteria were close to being met in the model of resident species richness at woodland points, but the plot of predicted values follows a trajectory of slowly accelerating loss of species at more urbanized locations with no evidence for a unimodal relationship with species richness peaking at intermediate levels of urbanization intensity (Appendix S3: Figure S1). All other species groupings (total species, native species, resident species, migrant non‐breeder, non‐forest species, and forest‐dependent species) exhibited linear declines in species richness as urbanization intensity increased (Table 2; Figure 2), with the exception of non‐native species richness, which exhibited a slight linear increase as urban intensification increased (Table 2; Figure 2c).

**TABLE 2:** Relationships between avian species richness and percentage impervious surface cover comparing between linear and quadratic models. Migrant non‐breeder species richness models (both location types) and non‐forest species richness at the woodland points were fitted with Poisson error structure (log link), non‐forest species richness at the randomized points were fitted with negative binomial models, and the rests were fitted with Gaussian error structure (identity link)

Response variable	Location type	Linear model			Quadratic model				
		Coefficient ± SE linear term	*p*‐value linear term	AIC_c_	Coefficient ± SE linear term	*p*‐value linear term	Coefficient ± SE quadratic term	*p*‐value quadratic term	AIC_c_
Total species richness	Randomized	−0.210 ± 0.016	<2.2e^−16^	945.74	−0.223 ± 0.063	0.001	1.3e^−4^ ± 0.001	0.838	947.81
	Woodland	−0.150 ± 0.013	<2.2e^−16^	869.90	−0.099 ± 0.049	0.044	−0.001 ± 5.0e^−4^	0.280	870.82
Native species richness	Randomized	−0.213 ± 0.016	<2.2e^−16^	950.31	−0.232 ± 0.064	3.88e^−4^	2.0e^−4^ ± 0.001	0.759	952.05
	Woodland	−0.158 ± 0.013	<2.2e^−16^	877.67	−0.107 ± 0.050	0.035	−0.001 ± 0.001	0.286	878.62
Non‐native species richness	Randomized	0.002 ± 0.001	0.039	133.14	0.007 ± 0.004	0.092	−5.1e^−5^ ± 4.3e^−5^	0.232	133.79
	Woodland	0.008 ± 0.002	5.4e^−6^	269.24	0.007 ± 0.007	0.270	7.0e^−6^ ± 6.7e^−5^	0.917	271.34
Resident species richness	Randomized	−0.192 ± 0.015	<2.2e^−16^	920.96	−0.181 ± 0.058	0.002	−1.2e^−4^ ± 0.001	0.842	923.03
	Woodland	−0.132 ± 0.012	<2.2e^−16^	854.07	−0.047 ± 0.046	0.309	−0.001 ± 4.7e^−4^	0.057	852.47
Migrant non‐breeder species richness	Randomized	−0.021 ± 0.004	8.9e^−9^	341.08	−0.022 ± 0.012	0.070	1.8e^−5^ ± 1.4e^−4^	0.900	343.14
	Woodland	−0.013 ± 0.003	1.9e^−6^	415.86	−0.027 ± 0.010	0.005	1.6e^−4^ ± 1.1e^−4^	0.137	415.77
Non‐forest species richness	Randomized	−0.010 ± 0.001	<2.2e^−16^	875.65	−0.008 ± 0.003	0.011	−2.4e^−5^ ± 3.2e^−5^	0.469	877.24
	Woodland	−0.006 ± 0.001	<2.2e^−16^	852.06	−0.004 ± 0.003	0.137	−2.2e^−5^ ± 2.7e^−5^	0.427	853.50
Forest‐dependent species richness	Randomized	−0.007 ± 0.001	2.0e^−10^	738.17	−0.001 ± 0.004	0.760	6.1e^−5^ ± 4.3e^−5^	0.154	738.20
	Woodland	−0.046 ± 0.007	1.1e^−9^	698.11	−0.002 ± 0.027	0.938	4.6e^−4^ ± 2.8e^−4^	0.100	697.44

### Objective 2: Does woodland cover moderate species richness–urbanization intensity relationships?

Species richness was consistently higher in woodland compared with randomized point counts for all species groups, except for non‐native species. Moreover, the interaction between type of point count (randomized or woodland) and urbanization intensity was significant in models of the species richness–urbanization intensity relationship for almost all species groups. This interaction term demonstrates that the slope of the linear changes in species richness along the urbanization gradient varies between randomized and woodland point count locations. Interaction terms were not significant in models of migrant non‐breeders and forest‐dependent species, indicating that these groups exhibited similar patterns of decline in richness along the urbanization gradient in randomized and woodland point count locations (Table 3). When interaction terms were significant, as urbanization intensity increased species richness almost invariably declined more slowly (i.e., had shallower gradients) in woodland points compared with the randomized ones; consequently, the gap in species richness between randomized and woodland points became larger as urbanization intensity increased (Table 3; Figure 2). Non‐native species exhibited the opposite pattern, with the difference in richness between randomized and woodland points narrowing as urbanization intensity increased (Table 3; Figure 2c).

**TABLE 3:** Coefficients and standard errors of the linear mixed models with Gaussian error structure for total bird species richness, native species richness, non‐native species richness, resident species richness, and forest‐dependent species richness and generalized linear mixed models with Poisson error structure for migrant non‐breeder species richness and non‐forest species richness as the response of percentage impervious surface cover (fixed effect) and its interaction term with location type (fixed effect), and grid ID as a random effect

Response variables	Intercept	Fixed effects		
		% impervious surface	Location type	Interaction term
Total species	34.079 ± 0.798	**−0.211 ± 0.014** **(*p* < 2.2e** ^**−16**^ **)**	1.302 ± 0.951 (*p* = 0.173)	**0.060 ± 0.017** **(*p* = 0.001)**
Native species	32.317 ± 0.813	**−0.214 ± 0.015** **(*p* < 2.2e** ^**−16**^ **)**	1.824 ± 0.959 (*p* = 0.059)	**0.054 ± 0.017** **(*p* = 0.002)**
Non‐native species	1.762 ± 0.078	0.003 ± 0.001 (*p* = 0.070)	**−0.522 ± 0.100** **(*p* = 5.0e** ^**−7**^ **)**	**0.005 ± 0.002** **(*p* = 0.005)**
Resident species	32.336 ± 0.741	**−0.194 ± 0.013** **(*p* < 2.2e** ^**−16**^ **)**	0.861 ± 0.890 (*p* = 0.335)	**0.060 ± 0.016** **(*p* = 2.7e** ^**−4**^ **)**
Migrant non‐breeders^a^	0.543 ± 0.157	**−0.020 ± 0.004** **(*p* = 4.0e** ^**−8**^ **)**	0.185 ± 0.194 (*p* = 0.342)	0.007 ± 0.005 (*p* = 0.137)
Non‐forest‐dependent species^a^	3.239 ± 0.040	**−0.010 ± 0.001** **(*p* < 2.2e** ^**−16**^ **)**	−0.080 ± 0.050 (*p* = 0.107)	**0.004 ± 0.001** **(*p* = 7.1e** ^**−5**^ **)**
Forest‐dependent species	9.834 ± 0.412	**−0.048 ± 0.007** **(*p* = 4.9e** ^**−10**^ **)**	**2.461 ± 0.483** **(*p* = 1.1e** ^**−6**^ **)**	0.004 ± 0.009 (*p* = 0.620)

*Note*: Significant effects (*p*‐values <0.05) are shown in bold.

a Models performed with Poisson error structure.

### Objective 3: Environmental characteristics and species richness in the randomized points

We modeled species richness for each of our various groups of species as a function of percentage impervious surface cover, distance to the nearest woodland, mean number of humans, tree species richness, total aboveground tree biomass, and aboveground biomass of large trees. Our model of non‐native species richness had very limited explanatory power (model *R* ^2^ = 6.5%) and did not contain any statistically significant predictors (Table 4). Explanatory power for all our other species richness models was substantially higher (model *R* ^2^ from 25.5% to 71.2%; Table 4), and percentage impervious surface cover and mean number of humans were consistently negatively associated with species richness (Table 4). The richness of forest‐dependent species was negatively associated with distance to the nearest woodland, and positively associated with tree species richness and above ground tree biomass (Table 4). Aboveground tree biomass was also positively associated with total species richness, native species richness and resident species richness (Table 4).

**TABLE 4:** Multiple regression models of bird species richness in randomized points as a function of percentage impervious surface, distance from the randomized plot to the nearest woodland (ln‐transformed), mean number of humans (ln‐transformed), tree species richness (ln‐transformed), total aboveground tree biomass (ln‐transformed), and aboveground biomass of large trees (ln‐transformed)

Response variable	Predictor											
	Impervious surface (%)		Distance to the nearest woodland		Mean no. humans		Tree species richness		Total aboveground tree biomass		Aboveground biomass of large trees	
	Coefficient ± SE (% partial *R* ^2^)	*p*	Coefficient ± SE (% partial *R* ^2^)	*p*	Coefficient ± SE (% partial *R* ^2^)	*p*	Coefficient ± SE (% partial *R* ^2^)	*p*	Coefficient ± SE (% partial *R* ^2^)	*p*	Coefficient ± SE (% partial *R* ^2^)	*p*
Total species (% *R* ^2^ = 71.19)	**−0.098 ± 0.019** **(16.01)**	**6.2e** ^**−7**^	0.109 ± 0.291 (0.10)	0.710	**−3.150 ± 0.397** **(30.62)**	**5.3e** ^**−13**^	−0.752 ± 0.829 (0.44)	0.427	**2.226 ± 0.829** **(4.80)**	**0.008**	−0.537 ± 0.490 (0.83)	0.275
Native species (% *R* ^2^ = 71.00)	**−0.100 ± 0.019** **(15.88)**	**6.9e** ^**−7**^	0.125 ± 0.296 (0.12)	0.674	**−3.209 ± 0.404** **(30.66)**	**5.1e** ^**−13**^	−0.675 ± 0.961 (0.34)	0.484	**2.175 ± 0.843** **(4.45)**	**0.011**	−0.501 ± 0.499 (0.70)	0.317
Non‐native species (% *R* ^2^ = 6.46)	0.001 ± 0.002 (0.40)	0.452	−0.016 ± 0.026 (0.27)	0.534	0.059 ± 0.036 (1.88)	0.100	−0.078 ± 0.085 (0.58)	0.361	0.051 ± 0.074 (0.32)	0.497	−0.036 ± 0.044 (0.46)	0.416
Resident species (% *R* ^2^ = 71.98)	**−0.088 ± 0.017** **(15.70)**	**8.1e** ^**−7**^	0.036 ± 0.263 (0.01)	0.892	**−2.920 ± 0.359** **(31.65)**	**1.8e** ^**−13**^	−0.431 ± 0.854 (0.18)	0.615	**1.931 ± 0.750** **(4.43)**	**0.011**	−0.407 ± 0.444 (0.58)	0.361
Migrant non‐breeders^a^ (% *R* ^2^ = 25.49)	**−0.012 ± 0.005** **(5.68)**	**0.015**	0.010 ± 0.074 (0.00)	0.893	**−0.255 ± 0.110** **(3.87)**	**0.021**	−0.281 ± 0.237 (1.09)	0.236	0.187 ± 0.200 (0.60)	0.349	−0.095 ± 0.121 (0.26)	0.434
Non‐forest species^a^ (% *R* ^2^ = 64.94)	**−0.004 ± 0.001** **(14.86)**	**1.6e** ^**−5**^	0.025 ± 0.016 (3.08)	0.115	**−0.166 ± 0.023** **(25.60)**	**3.8e** ^**−13**^	−0.089 ± 0.052 (2.17)	0.085	0.084 ± 0.045 (2.27)	0.060	−0.023 ± 0.027 (0.41)	0.387
Forest‐dependent species (% *R* ^2^ = 65.44)	**−0.022 ± 0.008** **(5.16)**	**0.006**	**−0.373 ± 0.123** **(6.08)**	**0.003**	**−0.591 ± 0.167** **(8.05)**	**0.001**	**0.998 ± 0.398** **(4.22)**	**0.013**	**0.773 ± 0.349** **(3.32)**	**0.028**	−0.184 ± 0.207 (0.55)	0.375

*Note*: Significant predictors (*p*‐values <0.05) are shown in bold.

a Models fitted using Poisson error structures.

### Objective 3: Environmental characteristics and species richness in the woodland points

Species richness for each of our various groups of species was modeled as a function of percentage impervious surface cover, size of the woodland, mean number of humans, tree species richness, total aboveground tree biomass, and aboveground biomass of large trees. Our models explained between ~15% and 60% of the variation in our response variables, with a noticeable improvement in explanatory capacity for non‐native species richness relative to that in the randomized points (Table 5). Again, the percentage of impervious surface cover was negatively associated with species richness for all our species groups except non‐native species richness, for which there was a marginally significant positive effect (Table 5). The mean number of humans at the point count locations was negatively associated with species richness for all species groups except non‐native species, for which there was a significant positive effect, and migrant non‐breeders (no significant effect; Table 5). The number of forest‐dependent species was positively associated with the size of the sampled woodland, tree species richness and above ground tree biomass (Table 5). The only other significant effects were negative impacts of woodland size on the number of non‐native species and negative effects of tree species richness on non‐forest species (Table 5).

**TABLE 5:** Multiple regression models of bird species richness in woodland points as a function of percentage impervious surface, size of the sampled woodland (ln‐transformed), mean number of humans (ln‐transformed), tree species richness (ln‐transformed), total aboveground tree biomass (ln‐transformed), and aboveground biomass of large trees (ln‐transformed)

Response variable	Predictor											
	Impervious surface (%)		Size of sampled woodland		Mean no. humans		Tree species richness		Total aboveground tree biomass		Aboveground biomass of large trees	
	Coefficient ± SE (% partial *R* ^2^)	*p*	Coefficient ± SE (% partial *R* ^2^)	*p*	Coefficient ± SE (% partial *R* ^2^)	*p*	Coefficient ± SE (% partial *R* ^2^)	*p*	Coefficient ± SE (% partial *R* ^2^)	*p*	Coefficient ± SE (% partial *R* ^2^)	*p*
Total species (% *R* ^2^ = 59.44)	**−0.107 ± 0.014** **(28.48)**	**4.8e** ^**−12**^	0.314 ± 0.349 (0.56)	0.370	**−2.180 ± 0.439** **(14.71)**	**1.9e** ^**−6**^	−0.740 ± 0.648 (0.91)	0.255	0.087 ± 0.604 (0.01)	0.886	0.163 ± 0.307 (0.20)	0.597
Native species (% *R* ^2^ = 61.80)	**−0.110 ± 0.014** **(29.25)**	**1.4e** ^**−12**^	0.477 ± 0.352 (1.27)	0.178	**−2.334 ± 0.443** **(16.27)**	**4.9e** ^**−7**^	−0.827 ± 0.653 (1.11)	0.208	0.240 ± 0.609 (0.11)	0.694	0.157 ± 0.310 (0.18)	0.615
Non‐native species (% *R* ^2^ = 29.29)	0.003 ± 0.002 (1.64)	0.122	**−0.164 ± 0.047** **(7.66)**	**0.001**	**0.155 ± 0.060** **(4.48)**	**0.011**	0.086 ± 0.088 (0.66)	0.329	−0.153 ± 0.082 (2.38)	0.064	0.006 ± 0.042 (0.02)	0.881
Resident species (% *R* ^2^ = 57.42)	**−0.089 ± 0.013** **(23.61)**	**4.2e** ^**−10**^	0.201 ± 0.327 (0.26)	0.539	**−2.244 ± 0.411** **(17.23)**	**2.1e** ^**−7**^	−0.582 ± 0.607 (0.64)	0.339	0.012 ± 0.565 (3.4e^−4^)	0.982	0.125 ± 0.288 (0.13)	0.666
Migrant non‐breeders^a^ (% *R* ^2^ = 16.75)	**−0.013 ± 0.003** **(9.76)**	**1.7e** ^**−4**^	0.093 ± 0.072 (1.08)	0.197	0.034 ± 0.106 (−7.1e^−5^)	0.748	−0.035 ± 0.144 (−0.11)	0.805	0.094 ± 0.131 (0.22)	0.472	0.009 ± 0.068 (0.05)	0.894
Non‐forest species^a^ (% *R* ^2^ = 50.91)	**−0.004 ± 0.001** **(19.72)**	**4.9e** ^**−7**^	−0.024 ± 0.021 (2.53)	0.251	**−0.098 ± 0.029** **(9.05)**	**0.001**	**−0.080 ± 0.038** **(3.88)**	**0.034**	−0.068 ± 0.035 (4.19)	0.052	0.023 ± 0.019 (0.85)	0.211
Forest‐dependent species (% *R* ^2^ = 54.77)	**−0.027 ± 0.007** **(9.77)**	**1.3e** ^**−4**^	**0.837 ± 0.168** **(14.82)**	**1.7e** ^**−6**^	**−0.737 ± 0.211** **(7.86)**	**0.001**	**0.865 ± 0.311** **(5.12)**	**0.006**	**1.258 ± 0.290** **(11.62)**	**2.7e** ^**−5**^	−0.242 ± 0.148 (1.84)	0.104

*Note*: Significant predictors (*p*‐values <0.05) are shown in bold.

a Models fitted using Poisson error structure.

### Objective 4: Environmental characteristics and community dissimilarity between randomized and woodland points

Jaccard's indices of dissimilarity in species composition between the randomized and woodland points for all bird species, natives, residents, and non‐consistently increased in less urbanized grid cells, when distance to the nearest woodland was greater, the sampled woodland was larger and there was a greater difference in mean numbers of people recorded at the two locations (Table 6). These models explained between ~18% and 25% of the variation in Jaccard's dissimilarity indices. Dissimilarity in species composition of forest‐dependent species assemblages increased significantly with distance to the nearest woodland and the difference in tree species richness between the randomized and woodland points (model *R* ^2^ = 24.0%; Table 6). No other predictor variables were significantly associated with the Jaccard's dissimilarity indices of any of our focal avian assemblages.

**TABLE 6:** Multiple regression models of Jaccard's dissimilarity index (%) for total bird species, native species, resident species, non‐forest species and forest‐dependent species in two sampling location (randomized and woodland points) as functions of percentage impervious surface, distance from the randomized plot to the nearest woodland (ln‐transformed) and to the sampled woodland, size of the sampled woodland, and absolute differences in habitat characteristics (number of humans (ln‐transformed), tree species richness (ln‐transformed), total aboveground tree biomass (ln‐transformed), and aboveground biomass of large trees (ln‐transformed))

Response variables	Predictor															
	Impervious surface (%)		Distance to nearest woodland		Distance to sampled woodland		Size of sampled woodland		Difference in no. humans		Difference in tree species richness		Difference in total AGB		Difference in AGB of large trees	
	Coefficient ± SE (% partial *R* ^2^)	*p*	Coefficient ± SE (% partial *R* ^2^)	*p*	Coefficient ± SE (% partial *R* ^2^)	*p*	Coefficient ± SE (% partial *R* ^2^)	*p*	Coefficient ± SE (% partial *R* ^2^)	*p*	Coefficient ± SE (% partial *R* ^2^)	*p*	Coefficient ± SE (% partial *R* ^2^)	*p*	Coefficient ± SE (% partial *R* ^2^)	*p*
Total species (% *R* ^2^ = 24.40)	**−0.151 ± 0.043** **(8.08)**	**0.001**	**2.533 ± 0.547** **(13.78)**	**6.3e** ^**−6**^	−3.26e^−4^ ± 0.007 (1.5e^−3^)	0.964	**2.908 ± 1.045** **(5.31)**	**0.006**	**3.485 ± 0.965** **(8.63)**	**4.2e** ^**−4**^	1.686 ± 1.331 (1.15)	0.207	−0.907 ± 0.892 (0.74)	0.311	0.252 ± 0.692 (0.10)	0.716
Native species (% *R* ^2^ = 25.74)	**−0.132 ± 0.044** **(6.19)**	**0.003**	**2.526 ± 0.548** **(13.36)**	**9.0e** ^**−6**^	3.10e^−4^ ± 0.007 (1.3e^−3^)	0.966	**2.584 ± 1.062** **(4.12)**	**0.016**	**4.035 ± 0.980** **(10.93)**	**6.6e** ^**−5**^	2.381 ± 1.352 (2.20)	0.080	−0.971 ± 0.906 (0.83)	0.285	0.107 ± 0.703 (0.02)	0.880
Resident species (% *R* ^2^ = 24.63)	**−0.149 ± 0.044** **(7.58)**	**0.001**	**2.645 ± 0.554** **(14.17)**	**4.6e** ^**−6**^	0.002 ± 0.007 (0.04)	0.805	**2.713 ± 1.075** **(4.41)**	**0.013**	**3.693 ± 0.992** **(9.12)**	**2.9e** ^**−4**^	1.821 ± 1.368 (1.27)	0.185	−1.274 ± 0.917 (1.38)	0.167	0.367 ± 0.711 (0.19)	0.607
Non‐forest species (% *R* ^2^ = 17.38)	**−0.166 ± 0.047** **(8.24)**	**0.001**	**1.425 ± 0.587** **(4.10)**	**0.016**	4.67e^−4^ ± 0.008 (2.6e^−3^)	0.953	**2.998 ± 1.138** **(4.79)**	**0.009**	**3.895 ± 1.051** **(9.05)**	**3.0e** ^**−4**^	−0.027 ± 1.449 (2.5e^−4^)	0.985	−1.643 ± 0.971 (2.03)	0.093	0.539 ± 0.753 (0.37)	0.475
Forest‐dependent species (% *R* ^2^ = 24.04)	−0.128 ± 0.071 (2.34)	0.071	**4.772 ± 0.880** **(17.55)**	**2.6e** ^**−7**^	−0.001 ± 0.012 (0.01)	0.899	3.011 ± 1.707 (2.20)	0.080	2.547 ± 1.576 (1.86)	0.108	**5.126 ± 2.173** **(3.87)**	**0.020**	0.546 ± 1.456 (0.10)	0.709	0.013 ± 1.130 (1.0e^−4^)	0.991

*Note*: Significant predictors (*p*‐values <0.05) are shown in bold. Non‐native species and migrant non‐breeder species categories were excluded from the analysis due to insufficient detection at each site.

## DISCUSSION

Urbanization is rapidly transforming Earth's land masses, with tropical regions typically experiencing faster urbanization rates than temperate ones (Seto et al., 2012). Urban development is therefore a major driver of the loss of tropical biodiverse habitats (McDonald et al., 2020; van Vliet, 2019) and the global biodiversity crisis (Aronson et al., 2014; McDonald et al., 2008), with urban areas often being hotspots for threatened species (Ives et al., 2016). Our study draws attention to key issues that enhance the understanding of biodiversity responses to urbanization including the form of tropical species richness–urbanization relationships, divergent responses across different types of species, how retention of woodland can moderate biodiversity responses to urban development, and the types of woodland that is most effective in supporting biodiversity. We discuss each of these issues in turn and provide recommendations for conservation action in rapidly urbanizing tropical regions:

### The importance of urban areas for forest‐dependent species and migrants

We did not detect any highly forest‐dependent species, that is, specialists that mainly occur in undisturbed forests and are rarely found in degraded forest habitats (as defined by BirdLife International (2019)), but documented 41 (28.9%) species with medium forest dependency, that is, those that are mainly found in undisturbed forest but also occur in forest edge and secondary forest. Many of these species were fairly widespread throughout our focal grid cells, being detected in at least one‐quarter of them, for example, Pink‐necked Green Pigeon *Treron vernans*, Brown‐throated Sunbird *Anthreptes malacensis* and Lineated Barbet *Megalaima lineata* (please refer to [https://doi.org/10.6084/m9.figshare.16557021] for more examples). Their presence within our study region demonstrate that some relatively specialized species can occur in urbanized regions helping to maintain a range of ecological functions such as pollination (Brown‐throated Sunbird) and seed‐dispersal (frugivores such as Pink‐necked Green Pigeon and Lineated Barbet). We also detected 36 migrant non‐breeder species (25.4% of our total) confirming the importance of considering urban areas when developing strategies to maintain bird migration along the East Asian–Australasian flyway (BirdLife International, 2015; Yong et al., 2015). In combination, these findings underscore the fact that urban areas must be considered when setting regional and global conservation agendas (Ives et al., 2016).

### Dominance of native species

Urban assemblages, including avian ones, are often considered to include a high proportion of established non‐native species (Lazarina et al., 2020; Marzluff, 2001). Yet, only a small proportion of species detected in our surveys (five of the 142 recorded species, 3.5%) were non‐native to the Bangkok region and, of these, only two species were widespread (Rock Pigeon *Columba livia* and Zebra Dove *Geopelia striata*). This is particularly surprising given that Bangkok is a major center for the captive bird trade (Chng & Eaton, 2016; Round, 1990), which is often assumed to increase the risk of accidental introductions (Reino et al., 2017). Notably, however, some of the widespread native species that we detected are widely considered to be dominant competitors that are non‐native invasive species in other regions, including the Common Myna *Acridotheres tristis* (94.7% occupancy; Colléony & Shwartz, 2020), Scaly‐breasted Munia *Lonchura punctulata* (80.0% occupancy; Conn et al., 2017), and Red Turtle Dove *Streptopelia tranquebarica* (82.7% occupancy; Yeo & Chia, 2010). Interspecific competition can play a key role in structuring urban bird communities (Martin & Bonier, 2018). It is therefore possible that the competitive abilities of these native species limit the extent to which non‐native species have become established in Bangkok. Such factors may contribute to the low occupancy rates of three of the five non‐native species detected in our survey (Rose‐ringed Parakeet *Psittacula krameri*, Alexandrine Parakeet *Palaeornis eupatria*, and Java Sparrow *Lonchura oryzivora*), especially as some of these are much more widespread in parts of their non‐native ranges, such as Rose‐ringed Parakeet *Psittacula krameri* (Pârâu et al., 2016). The later species is regarded as one of the 100 worst alien species in Europe (Brochier et al., 2010) and may in future expand throughout the Bangkok region threatening native cavity nesting species as is thought to be the case elsewhere in the Rose‐ringed Parakeet's non‐native range (Strubbe & Matthysen, 2009). Finally, and despite the low number of non‐native species, we found that the richness of non‐native species increased along the urbanization intensity gradient, contrasting with declines in species richness for all other groups, therefore providing support for conclusions that urban areas favor non‐native species (Lazarina et al., 2020; Tomasevic & Marzluff, 2017).

### The form of tropical species richness–urbanization intensity relationships

In temperate regions, avian species richness typically follows a hump‐shaped pattern along the urbanization intensity gradient, with richness peaking at intermediate levels of urbanization (Blair, 2004; Marzluff, 2005; Smith & Wachob, 2006; Vignoli et al., 2013). We found no evidence for such patterns in our tropical case study, with richness of all species groups (except non‐native species) peaking in the least urbanized parts of our urbanization gradient, and almost invariably declining in a linear manner as urbanization increased. These linear declines arise regardless of if surveys are conducted at randomized locations, or near the center of the largest patch of woodland habitat within each grid cell. Such linear species richness–urbanization intensity patterns have been documented in the relatively small number of similar studies conducted in urban regions, although not all have formally tested for alternative patterns (Chamberlain et al., 2017; Filloy et al., 2019; Leveau et al., 2017; Reis et al., 2012). While our sampling gradient did not extend into natural habitats (i.e., Khao Yai National Park, ~100 km from Bangkok center), doing so is unlikely to generate a unimodal richness–urban intensity gradient due to the extremely high avian richness of this location (Round et al., 2011). We therefore consider that the contrasting form of these relationships between tropical and temperate regions is likely to be a general pattern. In temperate regions habitat diversity often peaks at intermediate levels of urbanization intensity while the rural landscape is intensively used and consequently contains limited habitat heterogeneity, and this is considered to be the major mechanism driving the unimodal richness pattern (Tratalos et al., 2007). The negative linear pattern observed in our tropical case study might therefore be generated by a high diversity of habitats in the least urbanized parts of our gradient (i.e., interspersing of seminatural wetland, rice fields, orchards, and patches of secondary woodland, etc.), with habitat diversity gradually declining as urbanization increases. This might be a general driver of linear declines in species richness along urbanization gradients in tropical locations. In addition, it is plausible, albeit not unequivocal, that tropical regions contain a greater proportion of specialized species (Belmaker et al., 2012; Cirtwill et al., 2015). As urbanization selects against specialized species (Callaghan et al., 2019; Evans et al., 2011) linear declines in species richness along the urbanization gradient may be more likely to occur in tropical regions than temperate ones. This highlights the role of spatial patterns in local extinction rates in generating the form of species richness–urbanization intensity relationships (Marzluff, 2005).

### Moderating effects of woodland on the impacts of urbanization

Woodland survey points had higher species richness than the randomized points for almost all species groups. This is unsurprising given that other studies have demonstrated the importance of woodland in urban environments for enhancing the diversity of bird communities (Ferenc et al., 2014; Filloy et al., 2019; Pellissier et al., 2012). Our results regarding the impact of woodland on the richness of urban bird assemblages, however, go beyond confirming results of earlier studies in two important aspects. First, we find that non‐native species are an exception to this rule, and have lower species richness at the woodland points. This suggests that maintaining patches of more natural vegetation in urban environment could reduce the extent to which urban environments are prone to invasion by non‐native avian species. Second, we assess how woodland patches moderate changes in species richness along the urbanization gradient. For those species groups that decline in richness in response to urbanization we typically find shallower declines in species richness along the urbanization gradient at the woodland points. Consequently, the greatest gains in species richness from adding patches of woodland to the environment occur in the most urbanized sites. This has important implications for policies regarding habitat protection and restoration in urban environments as even small patches of woodland (mean woodland size in our most urbanized grid cells, that is, 90%–100% impervious surface cover) was 0.38 ± 0.21 (SE) ha (median = 0.13 ha) can deliver biodiversity benefits in intensely urbanized locations. This contrasts with the traditional dogma that small patches of natural habitat are likely to have little biodiversity value, although this view has recently been challenged (Wintle et al., 2019). While woodland did not moderate the effect of urbanization intensity on the richness of migrant non‐breeders and forest‐dependent species the beneficial impacts of woodland were maintained across the urbanization gradient, further suggesting that small patches of woodland surrounded by a highly urbanized matrix can provide biodiversity benefits. Such locations often represent the only greenspace in such areas and are therefore the last chance for conserving their remaining bird diversity (Savard et al., 2000; Soanes & Lentini, 2019). Larger patches of green space, especially of more natural vegetation types, should not be ignored in urban conservation initiatives (please refer to section “*Effects of spatial configuration and ecological characteristics of woodland patches*”).

### Human disturbance

Positive influences of human disturbance on non‐native species richness is probably driven by responses of two widespread non‐native commensal species (i.e., Rock Pigeon *Columba livia* and Zebra Dove *Geopelia striata*) that largely rely on human‐derived food resources (Round & Gardner, 2008). The richness of all other groups, except migrant non‐breeders in the woodland points, declined as human disturbance increased. Human disturbance was the predictor variable with the greatest explanatory power at the randomized points, and was the second most influential predictor variable (with regard to explanatory power) at the woodland points. Small numbers of other studies have also reported the negative impacts of human disturbance on urban bird assemblages (Jasmani et al., 2017; Kang et al., 2015), yet discussions of conservation actions in urban settings tend to focus on altering habitat types and management practices rather than grappling with human disturbance issues (e.g., Aronson et al., 2017). Our results therefore expose a trade‐off between the desire to manage urban green spaces in a manner that maximizes biodiversity and increasing residents' exposure to nature, which is likely to enhance their appreciation of biodiversity and the desire to protect it (Coldwell & Evans, 2017), alongside gaining well‐being benefits (Taylor & Hochuli, 2015), but at the risk of reducing avian biodiversity.

### Effects of spatial configuration and ecological characteristics of woodland patches

Randomized survey points that were further away from woodland had lower numbers of forest‐dependent species, but not species in other groups. Turnover in the composition of bird assemblages between paired randomized and woodland points also increased when randomized points were further away from woodland. It is therefore clear that close spatial proximity of woodland enables woodland specialists and, to a lesser extent, other species that utilize woodland to occupy the urban matrix. This is probably because the willingness to travel across the urban matrix varies between species, with forest‐dependent species particularly reluctant to cross gaps between suitable habitat patches (Watson et al., 2005). Larger woodlands increased the richness of forest‐dependent species, and increased the turnover in species composition (relative to random points) for all species groups. This highlights that larger urban woodlands support a relatively unique assemblage of species and therefore play an important role in maximizing urban avian diversity (Sorte et al., 2020).

Tree species richness and above ground biomass positively influenced the richness of forest‐dependent species in both the randomized and woodland survey locations, with above ground biomass also promoting higher richness of some other groups in the randomized points. Notably, the difference in tree species richness between the randomized and woodland points was positively associated with the turnover in species composition between these points. Our results therefore suggest that woodland patches with a more diverse tree flora and high aboveground biomass are likely to be most important in enhancing urban avian diversity. Our results therefore provide evidence‐based guidelines that urban woodland creation schemes should seek to plant a wide range of tree species, at relatively high densities. We found no explicit evidence that particularly large trees provided additional benefits, but this is probably a reflection of their rarity in the urban landscape, with just 2.9% of trees meeting our definition of large trees dbh ≥70 cm, and we do advocate that all large trees are protected within urban landscapes for their biodiversity benefits. Indeed, their rarity may partially explain why we did not detect any highly forest‐dependent species in our surveys.

## CONCLUSIONS AND CONSERVATION RECOMMENDATIONS

Our study adds to the growing but limited evidence that tropical avian assemblages exhibit linear declines in richness along the urbanization gradient, contrasting with the typical unimodal pattern in temperate regions with richness peaking at intermediate urbanization intensities. Potential mechanisms driving this contrast are differences between temperate and tropical regions in how habitat heterogeneity varies along the urbanization gradient, and a greater proportion of specialized species that are sensitive to urbanization in tropical regions. Moreover, we provide novel evidence that retaining patches of urban woodland can mitigate some of the adverse effects of the intensity of urbanization on species richness. For many species groups the benefits of woodland patches increase as urbanization intensifies even though such woodland patches are typically very small. We suggest four main recommendations for bird conservation in tropical urban regions that are also likely to benefit other taxa: (1) patches of seminatural habitat and areas with high levels of habitat diversity embedded within the urban matrix should be conserved, (2) increasing tree biomass and species diversity in urban woodlands will improve the habitat quality of wooded habitats for avian biodiversity, (3) woodland creation across the urbanization gradient, including in highly urbanized locations, even small wooded patches will be beneficial and the additional woodland should be well distributed throughout the urban area to minimize the effects of habitat isolation, and (4) managing human disturbance in some areas, especially those of high habitat quality, to minimize the adverse effects on urban bird populations while ensuring the benefits of connecting people to nature are realized in other locations. Achieving these goals will require contributions from urban planners and managers, landscape architects, and local residents alongside robust evaluation of the most effective policies to achieve these objectives.

## CONFLICT OF INTEREST

The authors declare no conflict of interest.

## Supporting information


Appendix S1

Appendix S2

Appendix S3

## Data Availability

Data (Thaweepworadej & Evans, 2021) are available on Figshare at https://doi.org/10.6084/m9.figshare.16557021.
