# Supplementary material for: Avian species richness and tropical urbanization gradients: Effects of woodland retention and human disturbance
Source: Ecol Appl. 2022 Jun 19;32(6):e2586. doi: 10.1002/eap.2586 (PMC9541691; doi:10.1002/eap.2586)
Supplement: Supplementary file 1 — Appendix S1 [file EAP-32-e2586-s001.pdf]

Supporting Information. Thaweepworadej, P. and K. L. Evans. 2022. Avian species richness and tropical urbanization gradients: Effects of woodland retention and human disturbance. *Ecological Applications*.

Appendix S1

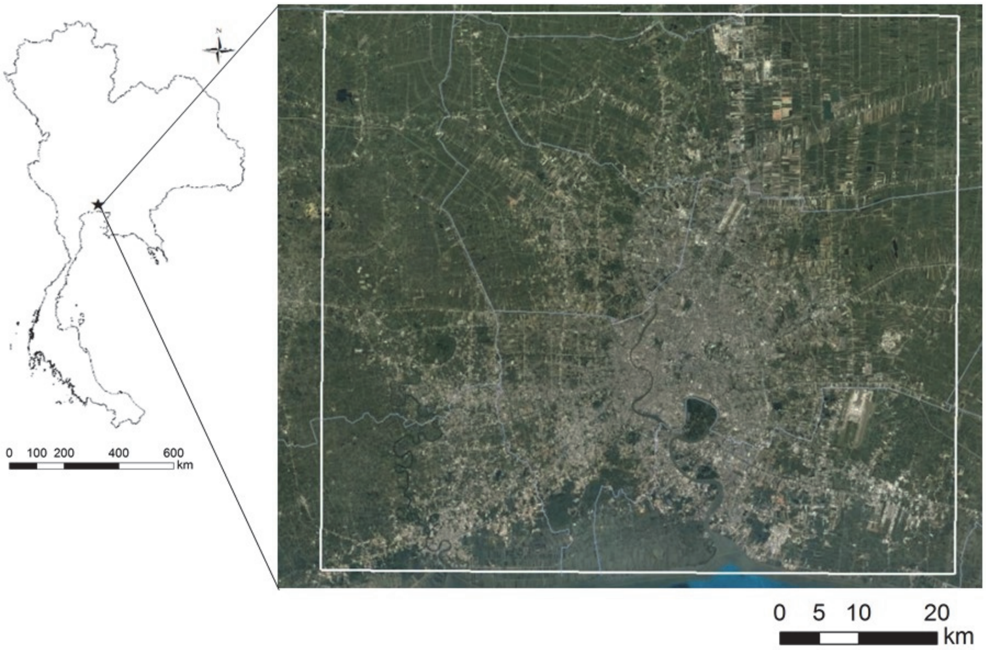

Figure S1: Map of Thailand showing the location of Bangkok and an inset map of the Bangkok region (using aerial imagery taken in March 2018). The rectangle with grey border delimits the 5,600 km^2 (70 km × 80 km rectangle) study region.
