# Supplementary material for: Avian species richness and tropical urbanization gradients: Effects of woodland retention and human disturbance
Source: Ecol Appl. 2022 Jun 19;32(6):e2586. doi: 10.1002/eap.2586 (PMC9541691; doi:10.1002/eap.2586)
Supplement: Supplementary file 2 — Appendix S2 [file EAP-32-e2586-s003.pdf]

Supporting Information. Thaweepworadej, P. and K. L. Evans. 2022. Avian species richness and tropical urbanization gradients: Effects of woodland retention and human disturbance. *Ecological Applications*.

Appendix S2

Table S1: Comparison of generalized linear models with Poisson (log link) or Gaussian (identity link) error structures, based on Akaike Information Criterion values adjusted for small sample size (AICc), for i) linear species richness-urbanization intensity models, ii) quadratic species richness-urbanization intensity models, iii) a mixed effects model of species richness as a function of survey point type, percentage impervious surface cover and their interaction, and iv) a full model with a complete suite of predictor variables describing landscape characteristics and ecological features. Mold indicates models with the lowest AICc for each comparison.

Response variable	Location type	AICc values							
		Linear urbanization model		Quadratic urbanization model		Mixed effects model		Full model	
		Poisson	Gaussian	Poisson	Gaussian	Poisson	Gaussian	Poisson	Gaussian
Total species richness	Randomized	960.20	**945.74**	960.19	**947.81**	1803.58	**1778.18**	888.96	**882.91**
	Woodland	881.20	**869.90**	880.82	**870.82**			869.67	**847.57**
Native species richness	Randomized	973.52	**950.31**	973.10	**952.33**	1813.94	**1787.65**	892.56	**888.20**
	Woodland	884.99	**877.67**	884.01	**878.62**			867.24	**869.67**
Non-native species richness	Randomized	398.21	**133.14**	396.62	**133.79**	777.06	**420.07**	406.42	**159.63**
	Woodland	391.62	**269.24**	393.70	**271.34**			396.31	**249.14**
Resident species richness	Randomized	933.79	**920.96**	932.96	**923.03**	1766.54	**1735.34**	868.36	**852.79**
	Woodland	868.65	**854.07**	866.39	**852.47**			856.45	**827.93**
Migrant non-breeder species richness	Randomized	**341.08**	400.97	**343.14**	400.53	**740.28**	841.30	**341.12**	401.64
	Woodland	**415.86**	462.19	**415.77**	459.38			**423.02**	469.40
Non-forest species richness	Randomized	**878.02**	890.23	**879.43**	891.75	**1681.70**	1698.16	**825.77**	845.74
	Woodland	**852.06**	863.23	**853.50**	865.32			**825.74**	833.17
Forest-dependent species richness	Randomized	**738.17**	738.39	**738.20**	739.75	1414.55	**1387.43**	660.92	**632.83**
	Woodland	709.36	**698.11**	709.02	**697.44**			684.30	**627.74**

Table S2: Overdispersion test (DHARMa package) results (dispersion parameter and *P*-value) for response variables that were fitted using Poisson error structure (see Table S1). Response variables with significant overdispersion (in bold) are modelled using negative binomial model.

Response variable	Location type	Overdispersion test results							
		Linear urbanization model		Quadratic urbanization model		Mixed effects model		Full model	
		Dispersion	*P*	Dispersion	*P*	Dispersion	*P*	Dispersion	*P*
Migrant non-breeder species richness	Randomized	1.002	0.480	1.002	0.476	0.842	0.904	0.932	0.636
	Woodland	0.997	0.472	0.991	0.508			0.976	0.560
Non-forest species richness	Randomized	**1.284**	**0.020**	**1.293**	**0.016**	0.908	0.864	0.882	0.848
	Woodland	1.000	0.488	0.996	0.496			0.730	0.996
Forest-dependent species richness	Randomized	1.027	0.396	0.987	0.528	-	-	-	-

Table S3: Moran I's test results of generalized linear models for bird species richness with percentage impervious surface cover (linear and quadratic models) in randomized and woodland points and generalized linear mixed models for bird species richness with percentage impervious surface cover and interaction term between percentage impervious surface cover and types of survey location (i.e. randomized and woodland points). Models with significant spatial autocorrelation are shown in bold.

Response variable	Location type	Moran I's test results					
		Linear urbanization model		Quadratic urbanization model		Mixed effects model	
		Observed	*P*	Observed	*P*	Observed	*P*
Total species richness	Randomized	-0.005	0.605	-0.005	0.574	-0.003	0.842
	Woodland	**-0.016**	**0.016**	**-0.015**	**0.028**		
Native species richness	Randomized	-0.008	0.785	-0.007	0.839	-0.003	0.837
	Woodland	-0.008	0.785	-0.011	0.230		
Non-native species richness	Randomized	-0.008	0.824	-0.006	0.929	**-0.008**	**0.025**
	Woodland	**-0.020**	**$3.2e^{-4}$**	**-0.020**	**$3.3e^{-4}$**		
Resident species richness	Randomized	-0.003	0.393	-0.004	0.413	-0.003	0.971
	Woodland	**-0.019**	**0.002**	**-0.016**	**0.011**		
Migrant non-breeder species richness	Randomized	-0.006	0.904	-0.006	0.891	**-0.009**	**0.006**
	Woodland	-0.009	0.554	-0.008	0.680		
Non-forest species richness	Randomized	-0.004	0.494	-0.005	0.562	**-0.007**	**0.040**
	Woodland	**-0.022**	**$3.0e^{-5}$**	**-0.018**	**$4.2e^{-5}$**		
Forest-dependent species richness	Randomized	-0.006	0.901	-0.008	0.758	-0.003	0.896
	Woodland	-0.011	0.266	-0.010	0.355		

Table S4: Comparison of parameter coefficients and standard errors of a) generalized linear models for bird species richness with percentage impervious surface cover (including linear and quadratic models) in the woodland points and b) generalized linear mixed models for bird species richness with percentage impervious surface cover and interaction term between percentage impervious surface cover and location types where there is evidence of significant spatial autocorrelation (Table S3) with and without taking spatial correlation structure into account by using generalized linear mixed model approach. Models were fitted with Gaussian error structure (identity link) for total species richness, non-native species richness, and resident species richness, and with Poisson error structure (log link) for non-forest species richness.

a)

Response variable	Model type	Linear urbanization model			Quadratic urbanization model				
		Intercept	Coeff ± SE	*P*	Intercept	Coeff ± SE (linear term)	*P* (linear term)	Coeff ± SE (quadratic term)	*P* (quadratic term)
Total species richness	Spatial	34.916 ± 0.770	-0.139 ± 0.014	$<2.2e^{-16}$	34.716 ± 1.042	-0.125 ± 0.050	0.014	$-1.6e^{-4} \pm 0.001$	0.763
	Non-spatial	35.323 ± 0.685	-0.150 ± 0.013	$<2.2e^{-16}$	34.539 ± 0.995	-0.099 ± 0.049	0.044	$-0.001 \pm 5.0e^{-4}$	0.280
Non-native species richness	Spatial	1.213 ± 0.092	0.008 ± 0.002	$<2.2e^{-16}$	1.223 ± 0.135	0.007 ± 0.007	0.270	$7.0e^{-6} \pm 6.7e^{-5}$	0.917
	Non-spatial	1.213 ± 0.092	0.008 ± 0.002	$5.4e^{-6}$	1.223 ± 0.135	0.007 ± 0.007	0.270	$7.0e^{-6} \pm 6.7e^{-5}$	0.917
Resident species richness	Spatial	32.536 ± 0.768	-0.114 ± 0.013	$<2.2e^{-16}$	31.900 ± 0.997	-0.069 ± 0.047	0.147	$-0.001 \pm 4.9e^{-4}$	0.298
	Non-spatial	33.119 ± 0.649	-0.132 ± 0.012	$<2.2e^{-16}$	31.813 ± 0.936	-0.047 ± 0.046	0.309	$-0.001 \pm 4.7e^{-4}$	0.057
Non-forest species richness	Spatial	3.115 ± 0.042	-0.005 ± 0.001	$<2.2e^{-16}$	3.117 ± 0.053	-0.005 ± 0.003	0.064	$2.0e^{-6} \pm 2.7e^{-5}$	0.943
	Non-spatial	3.151 ± 0.035	-0.006 ± 0.001	$<2.2e^{-16}$	3.122 ± 0.050	-0.004 ± 0.003	0.137	$2.2e^{-5} \pm 2.7e^{-5}$	0.427

b)

Response variable	Model type	Intercept	Fixed effects					
			% impervious surface		Location type		Interaction term	
			Coeff ± SE	*P*	Coeff ± SE	*P*	Coeff ± SE	*P*
Non-native species richness	Spatial	1.762 ± 0.078	0.003 ± 0.001	0.069	-0.522 ± 0.111	$2.4e^{-6}$	0.005 ± 0.002	0.011
	Non-spatial	1.762 ± 0.078	0.003 ± 0.001	0.070	-0.522 ± 0.100	$5.0e^{-7}$	0.005 ± 0.002	0.005
Migrant non-breeder species richness	Spatial	0.542 ± 0.179	-0.020 ± 0.004	$7.7e^{-7}$	0.229 ± 0.238	0.336	0.005 ± 0.005	0.280
	Non-spatial	0.543 ± 0.157	-0.020 ± 0.004	$4.0e^{-8}$	0.185 ± 0.194	0.342	0.007 ± 0.005	0.137
Non-forest species richness	Spatial	3.230 ± 0.054	-0.009 ± 0.001	$< 2.2e^{-16}$	-0.081 ± 0.073	0.269	0.004 ± 0.001	0.001
	Non-spatial	9.834 ± 0.412	-0.010 ± 0.001	$< 2.2e^{-16}$	-0.080 ± 0.050	0.107	0.004 ± 0.001	$7.1e^{-5}$

Table S5: Variance Inflation Factors (VIFs) values of multiple regression models for bird species richness with landscape and ecological attributes (separately for randomized and woodland points), and species turnover (Jaccard's dissimilarity index; JDI) of bird community in randomized and woodland points revealed no serious multicollinearity amongst our models (VIF < 10).

Predictor variables	VIFs
*Bird species richness at the randomized location*s	
% impervious surface cover of grid cell	2.131
Distance from the randomized plot to the nearest woodland	1.996
Mean number of humans	2.136
Tree species richness	4.159
Total aboveground tree biomass	6.343
Aboveground tree biomass of large trees	1.965
Bird species richness at the woodland locations	
% impervious surface cover of grid cell	1.549
Size of the sampled woodland	1.314
Mean number of humans	1.591
Tree species richness	1.331
Total aboveground tree biomass	2.044
Aboveground tree biomass of large trees	1.805
Species turnover (JDI) between randomized and woodland locations	
% impervious surface cover of grid cell	1.928
Distance from the randomized plot to the nearest woodland	1.152
Distance from the randomized plot to the sampled woodland	1.062
Size of the sampled woodland	1.289
Absolute difference in mean number of humans	1.892
Absolute difference in tree species richness	1.154
Absolute difference in total aboveground tree biomass	1.248
Absolute difference in aboveground tree biomass of large trees	1.194

Table S6: Moran's I test results of multiple regression models for bird species richness in randomized and woodland points, and species turnover (Jaccard's dissimilarity index; JDI) of bird community in randomized and woodland points as a function of landscape characteristics and ecological features (Table 1 in the main manuscript). Models for species richness of migrant non-breeder and non-forest species were fitted with Poisson error structure and the rest with Gaussian error structure. Models with significant spatial autocorrelation are shown in bold.

Response variable	Moran's I test					
	Randomized		Woodland		JDI	
	Observed	*P*	Observed	*P*	Observed	*P*
Total species richness	**0.021**	**0.011**	**0.027**	**0.002**	-0.020	0.821
Native species richness	**0.033**	**$2.8e^{-4}$**	**0.019**	**0.015**	-0.023	0.867
Non-native species richness	0.003	0.380	**0.029**	**0.001**	-	-
Resident species richness	**0.017**	**0.031**	**0.024**	**0.004**	-0.017	0.769
Migrant non-breeder species richness*	**-0.016**	**0.037**	0.006	0.251	-	-
Non-forest species richness*	-0.011	0.094	**0.028**	**0.001**	-0.010	0.577
Forest-dependent species richness	-0.012	0.603	-0.001	0.621	-0.009	0.561

Table S7: Comparison of parameter coefficients and standard errors of species richness in a) randomized and b) woodland points as functions of ecological features where there is evidence of significant spatial autocorrelation (Table S6) with and without taking spatial correlation structure into account by using generalized linear mixed model approach. Models were fitted with Gaussian error structure (identity link) for total species richness, native species richness, resident species richness, and forest-dependent species richness and with Poisson error structure (log link) for migrant non-breeder species richness.

a)

Response variable	Model type	% Impervious surface		Size of sampled woodland		Mean number of humans		Tree species richness		Total aboveground tree biomass		Aboveground biomass of large trees	
		Coeff ± SE	*P*	Coeff ± SE	*P*	Coeff ± SE	*P*	Coeff ± SE	*P*	Coeff ± SE	*P*	Coeff ± SE	*P*
Total species richness	Spatial	-0.097 ± 0.019	$<2.2e^{-16}$	0.106 ± 0.289	0.714	-3.162 ± 0.396	$<2.2e^{-16}$	-0.812 ± 0.946	0.392	2.295 ± 0.932	0.007	-0.597 ± 0.493	0.228
	Non-spatial	-0.098 ± 0.019	$6.2e^{-7}$	0.109 ± 0.291	0.710	-3.150 ± 0.397	$5.3e^{-13}$	-0.752 ± 0.944	0.427	2.226 ± 0.829	0.008	-0.537 ± 0.490	0.276
Native species richness	Spatial	-0.099 ± 0.019	$<2.2e^{-16}$	0.122 ± 0.295	0.680	-3.221 ± 0.403	$<2.2e^{-16}$	-0.733 ± 0.963	0.448	2.242 ± 0.847	0.009	-0.559 ± 0.501	0.267
	Non-spatial	-0.100 ± 0.019	$6.9e^{-7}$	0.125 ± 0.296	0.673	-3.209 ± 0.404	$5.1e^{13}$	-0.675 ± 0.961	0.484	2.175 ± 0.843	0.011	-0.501 ± 0.499	0.317
Resident species richness	Spatial	-0.087 ± 0.017	$<2.2e^{-16}$	0.034 ± 0.263	0.896	-2.924 ± 0.359	$<2.2e^{-16}$	-0.461 ± 0.856	0.591	1.967 ± 0.752	0.010	-0.435 ± 0.445	0.330
	Non-spatial	-0.088 ± 0.017	$8.1e^{-7}$	0.036 ± 0.263	0.892	-2.920 ± 0.359	$1.8e^{-13}$	-0.431 ± 0.854	0.615	1.931 ± 0.750	0.011	-0.407 ± 0.444	0.361
Migrant non-breeder species richness	Spatial	-0.012 ± 0.005	0.018	0.010 ± 0.074	0.897	-0.258 ± 0.110	0.021	-0.288 ± 0.237	0.227	0.194 ± 0.200	0.333	-0.103 ± 0.122	0.398
	Non-spatial	-0.012 ± 0.005	0.015	0.010 ± 0.074	0.893	-0.255 ± 0.110	0.021	-0.281 ± 0.237	0.236	0.187 ± 0.200	0.349	-0.095 ± 0.121	0.211

b)

Response variable	Model type	% Impervious surface		Size of sampled woodland		Mean number of humans		Tree species richness		Total aboveground tree biomass		Aboveground biomass of large trees	
		Coeff ± SE	*P*	Coeff ± SE	*P*	Coeff ± SE	*P*	Coeff ± SE	*P*	Coeff ± SE	*P*	Coeff ± SE	*P*
Total species richness	Spatial	-0.098 ± 0.015	$<2.2e^{-16}$	0.332 ± 0.339	0.329	-2.124 ± 0.422	$<2.2e^{-16}$	-0.977 ± 0.631	0.124	0.463 ± 0.583	0.429	0.129 ± 0.296	0.663
	Non-spatial	-0.107 ± 0.014	$4.8e^{-12}$	0.314 ± 0.349	0.370	-2.180 ± 0.439	$1.9e^{-6}$	-0.740 ± 0.648	0.255	0.087 ± 0.604	0.886	0.163 ± 0.307	0.597
Native species richness	Spatial	-0.110 ± 0.014	$<2.2e^{-16}$	0.478 ± 0.352	0.177	-2.333 ± 0.443	$<2.2e^{-16}$	-0.825 ± 0.653	0.209	0.239 ± 0.609	0.696	0.157 ± 0.310	0.613
	Non-spatial	-0.110 ± 0.014	$2.2e^{-12}$	0.477 ± 0.352	0.178	-2.334 ± 0.443	$4.9e^{-7}$	-0.827 ± 0.653	0.208	0.240 ± 0.609	0.694	0.157 ± 0.310	0.615
Non -native species richness	Spatial	0.003 ± 0.002	0.123	-0.164 ± 0.047	0.001	0.155 ± 0.060	0.011	0.086 ± 0.088	0.329	-0.153 ± 0.082	0.064	0.006 ± 0.042	0.881
	Non-spatial	0.003 ± 0.002	0.122	-0.164 ± 0.047	0.001	0.155 ± 0.060	0.011	0.086 ± 0.088	0.329	-0.153 ± 0.082	0.064	0.006 ± 0.042	0.881
Resident species richness	Spatial	-0.089 ± 0.013	$<2.2e^{-16}$	0.201 ± 0.327	0.539	-2.244 ± 0.411	$<2.2e^{-16}$	-0.582 ± 0.607	0.339	0.012 ± 0.565	0.982	0.125 ± 0.288	0.666
	Non-spatial	-0.089 ± 0.013	$4.2e^{-10}$	0.201 ± 0.327	0.539	-2.244 ± 0.411	$2.1e^{-7}$	-0.582 ± 0.607	0.339	0.012 ± 0.565	0.982	0.125 ± 0.288	0.666
Non-forest species richness	Spatial	-0.004 ± 0.001	$<2.2e^{-16}$	-0.021 ± 0.017	0.235	-0.091 ± 0.023	$1.0e^{-4}$	-0.087 ± 0.032	0.006	-0.050 ± 0.029	0.083	0.024 ± 0.015	0.122
	Non-spatial	-0.004 ± 0.001	$4.9e^{-7}$	-0.024 ± 0.021	0.251	-0.098 ± 0.029	0.001	-0.080 ± 0.038	0.034	-0.068 ± 0.035	0.052	0.023 ± 0.019	0.211

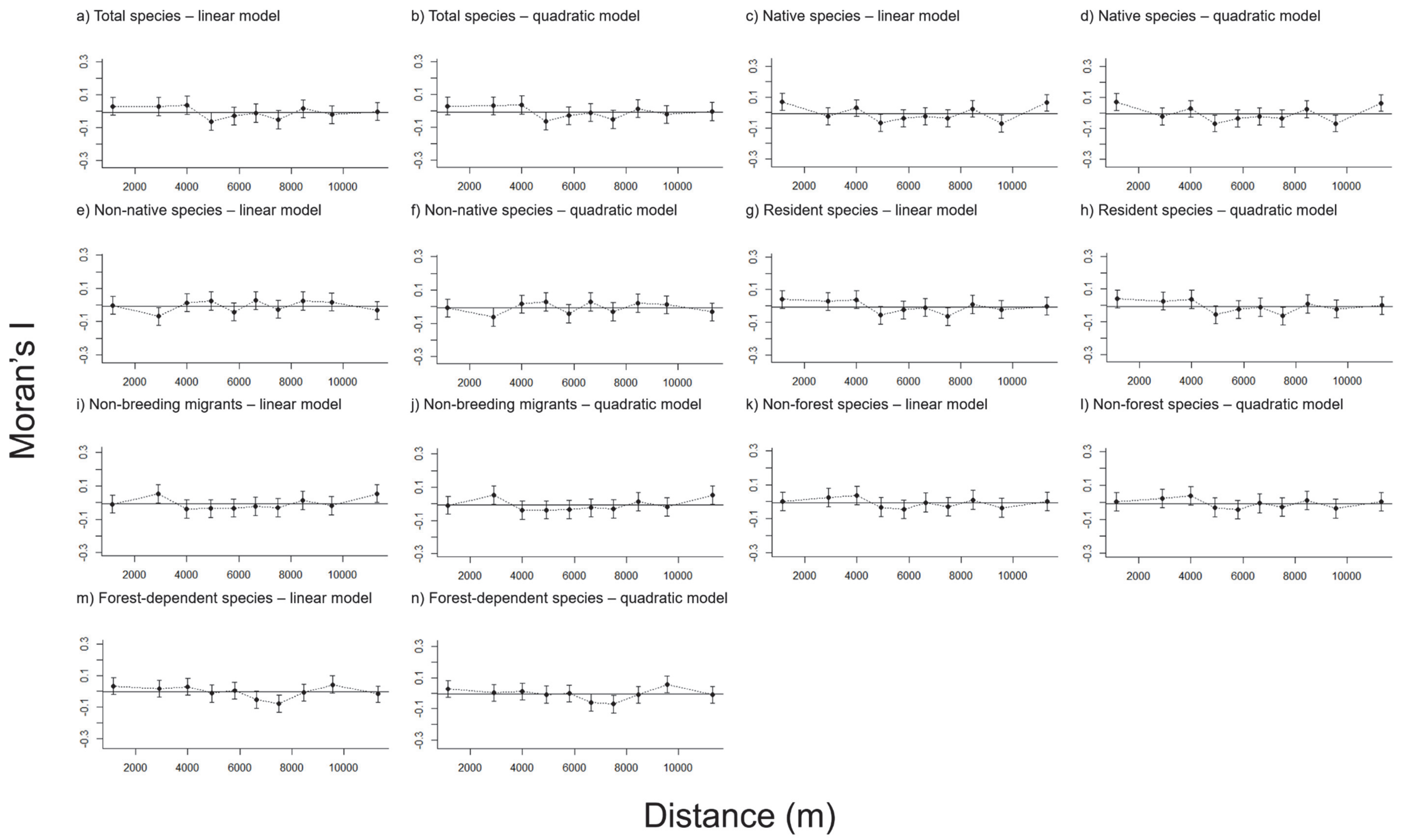

Figure S1: Correlograms of Moran's I values against lag distances (implemented using letsR package in R) for models of total species, native species, non-native species, resident species, migrant non-breeder, non-forest, and forest-dependent species richness at the randomized points as function of percentage impervious surface cover (including linear and quadratic models).

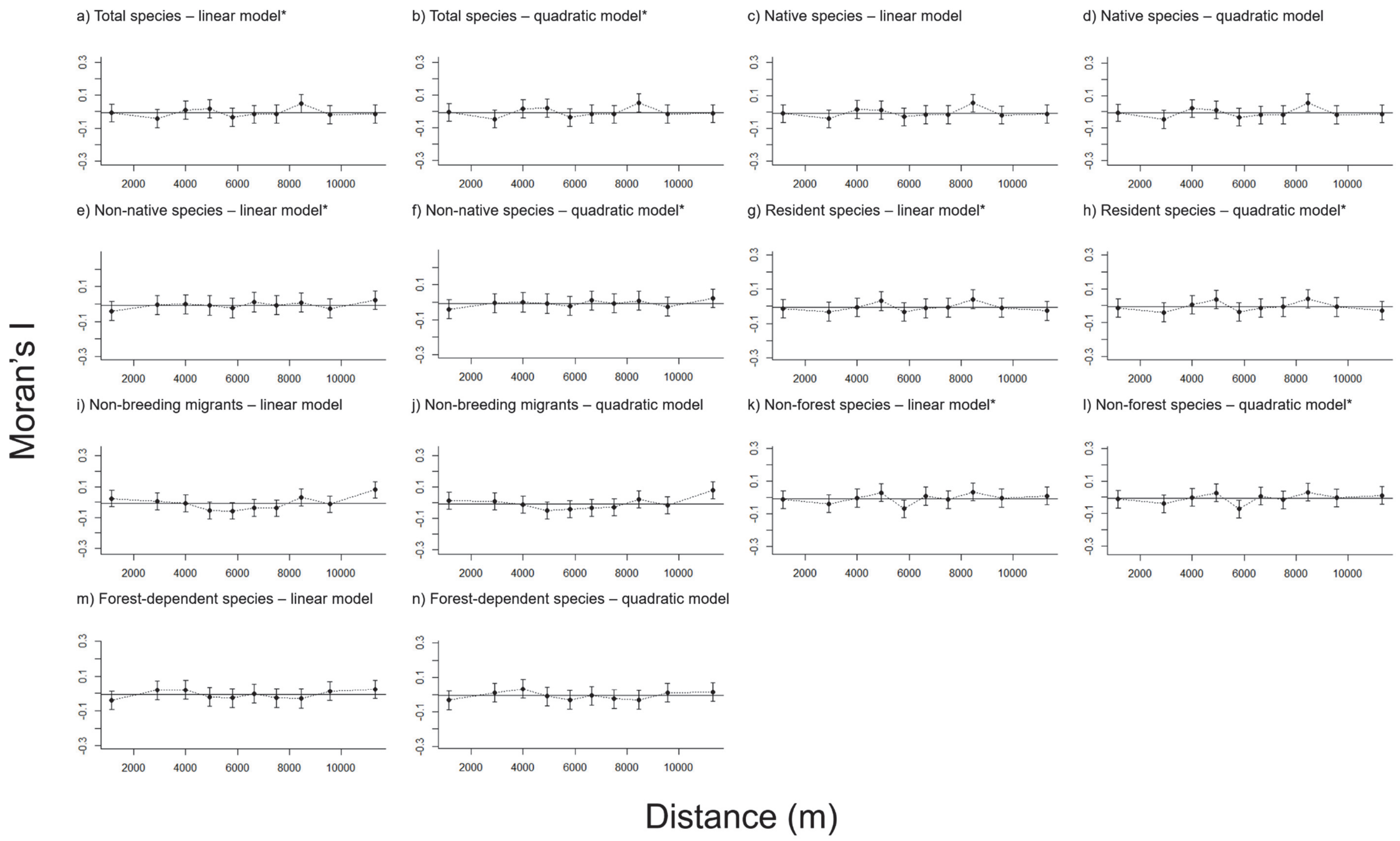

Figure S2: Correlograms of Moran's I values against lag distances (implemented using letsR package in R) for models of total species, native species, non-native species, resident species, migrant non-breeder, non-forest, and forest-dependent species richness at the woodland points as function of percentage impervious surface cover (including linear and quadratic models). Asterisks indicate model with significant spatial autocorrelation (detected using Moran's I test via ape package in R).

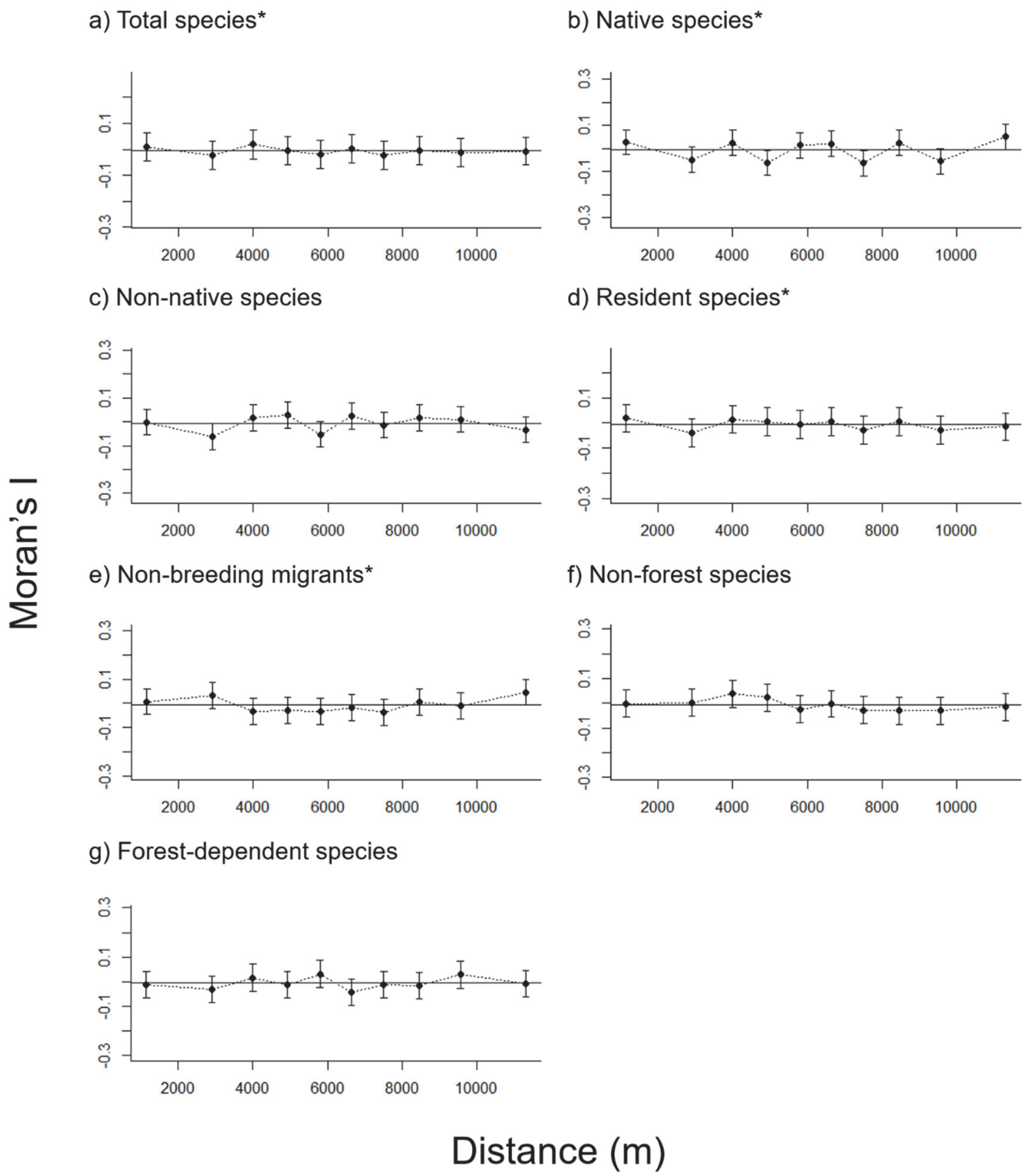

Figure S3: Correlograms of Moran's I against lag distances (implemented using letsR package in R) for models of total species, native species, non-native species, resident species, non-breeding migrants, non-forest, and forest-dependent species richness at the randomized points as function of percentage impervious surface cover, distance to the nearest woodland (ln-transformed), mean number of humans (ln-transformed), tree species richness (ln-transformed), total aboveground tree biomass (ln-transformed), and aboveground biomass of large trees (ln-transformed). Asterisks indicate model with significant spatial autocorrelation (detected using Moran's I test via ape package in R).

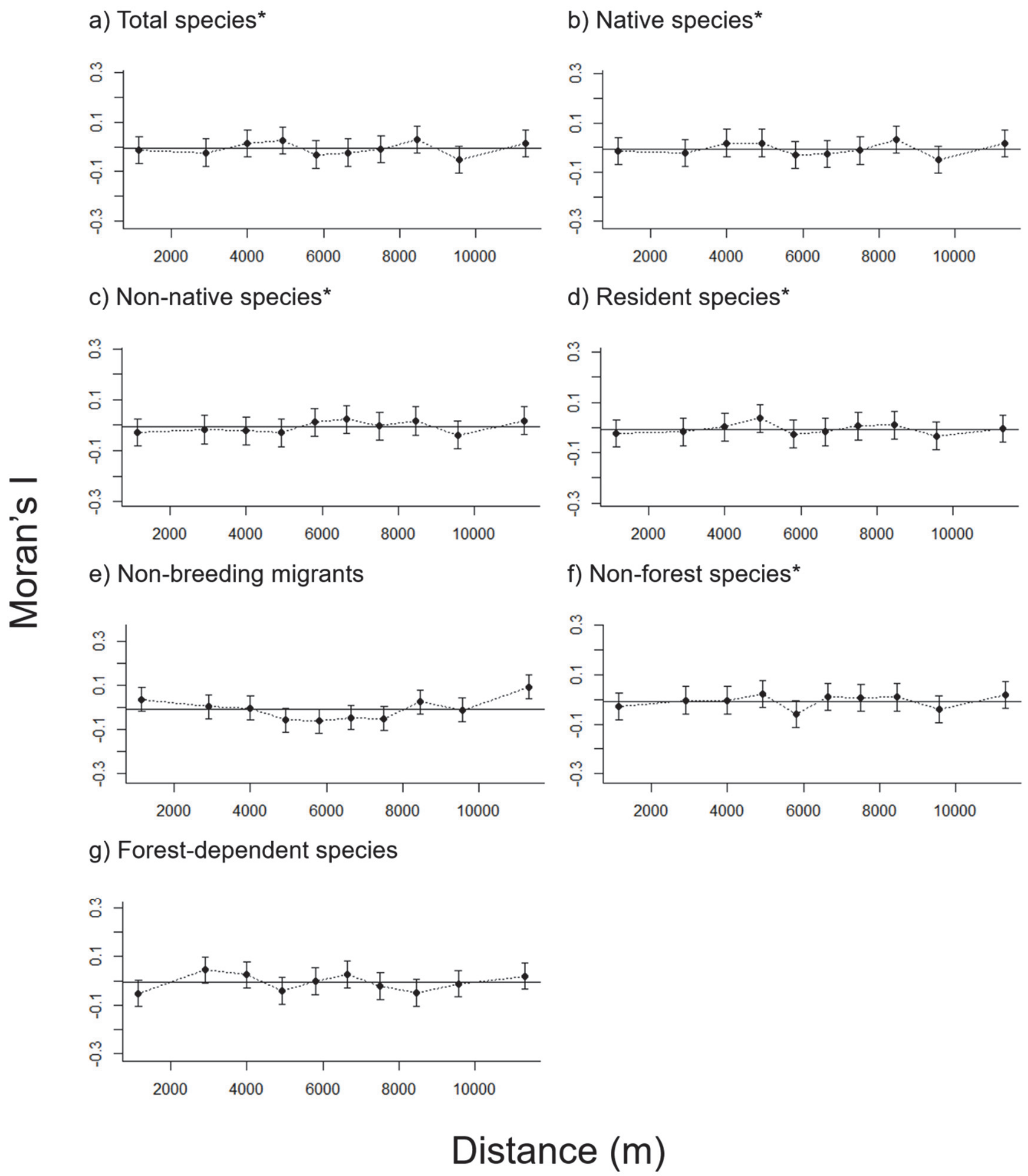

Figure S4: Correlograms of Moran's I against lag distances (implemented using letsR package in R) for models of total species, native species, non-native species, resident species, non-breeding migrants, non-forest, and forest-dependent species richness at the woodland points as function of percentage impervious surface cover, size of the sampled woodland, mean number of humans (ln-transformed), tree species richness (ln-transformed), total aboveground tree biomass (ln-transformed), and aboveground biomass of large trees (ln-transformed). Asterisks indicate model with significant spatial autocorrelation (detected using Moran's I test via ape package in R).

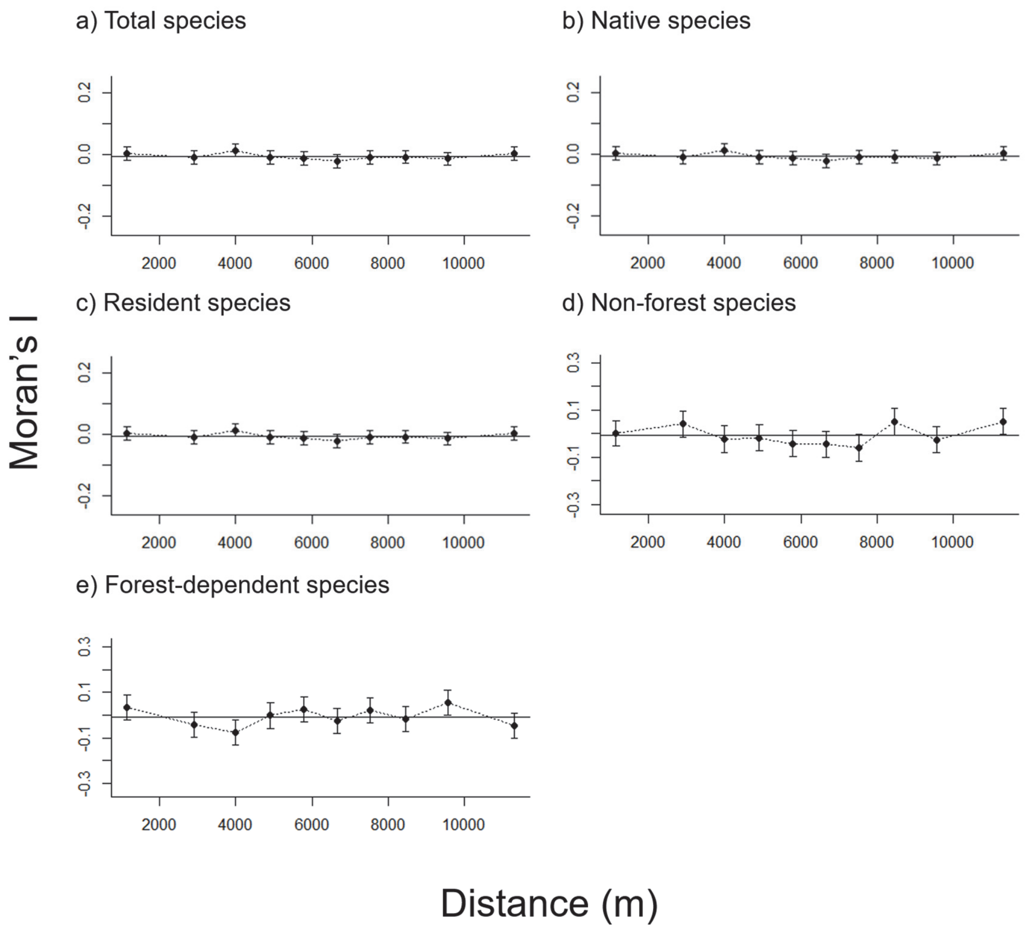

Figure S5: Correlograms of Moran's I against lag distances (implemented using letsR package in R) for models of Jaccard's dissimilarity index for total species, native species, resident species, non-forest, and forest-dependent species richness as function of percentage impervious surface cover, distance from randomized plot to the nearest woodland (ln-transformed) and to the sampled woodland, size of the sample woodland, and absolute differences in habitat characteristics (i.e. mean number of humans (ln-transformed), tree species richness (ln-transformed), total aboveground tree biomass (ln-transformed), and aboveground biomass of large trees (ln-transformed)).
