# Supplementary material for: Avian species richness and tropical urbanization gradients: Effects of woodland retention and human disturbance
Source: Ecol Appl. 2022 Jun 19;32(6):e2586. doi: 10.1002/eap.2586 (PMC9541691; doi:10.1002/eap.2586)
Supplement: Supplementary file 3 — Appendix S3 [file EAP-32-e2586-s002.pdf]

Supporting Information. Thaweepworadej, P. and K. L. Evans. 2022. Avian species richness and tropical urbanization gradients: Effects of woodland retention and human disturbance. *Ecological Applications*.

Appendix S3

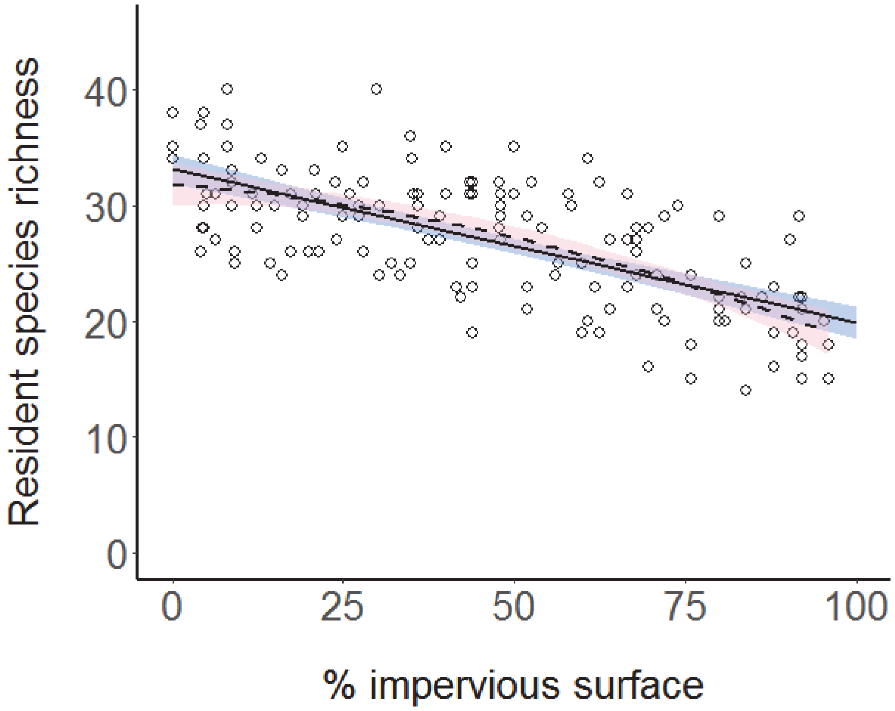

Figure S1: The urbanisation gradient for resident species richness at wooded survey points. Fitted lines indicate predicted values comparing between a linear model (solid line and blue shading) and a quadratic model (dashed line and pink shading); shading indicates 95% confidence intervals.
